# Developing an enhanced chimeric permuted intron-exon system for circular RNA therapeutics

**DOI:** 10.7150/thno.98214

**Published:** 2024-09-09

**Authors:** Lei Wang, Chunbo Dong, Weibing Zhang, Xu Ma, Wei Rou, Kai Yang, Tong Cui, Shaolong Qi, Lijun Yang, Jun Xie, Guocan Yu, Lianqing Wang, Xiaoyuan Chen, Zhida Liu

**Affiliations:** 1 MOE Key Laboratory of Coal Environmental Pathogenicity and Prevention, Shanxi Medical University, Taiyuan 030001, China.; 2 Shanxi Academy of Advanced Research and Innovation, Taiyuan 030032, China.; 3 Department of Biochemistry and Molecular Biology, Shanxi Key Laboratory of Birth Defect and Cell Regeneration, Shanxi Medical University, Taiyuan 030001, China.; 4 College of Veterinary Medicine, Shanxi Agricultural University, Jinzhong 030801, China.; 5 Key Laboratory of Bioorganic Phosphorus Chemistry & Chemical Biology, Department of Chemistry, Tsinghua University, Beijing 100084, China.; 6 Center of Translational Medicine, Zibo Central Hospital, Zibo 255036, China.; 7 Departments of Diagnostic Radiology, Surgery, Chemical and Biomolecular Engineering, and Biomedical Engineering, Yong Loo Lin School of Medicine and College of Design and Engineering, National University of Singapore, Singapore 119074, Singapore.; 8 Clinical Imaging Research Centre, Centre for Translational Medicine, Yong Loo Lin School of Medicine, National University of Singapore, Singapore 117599, Singapore.; 9 Nanomedicine Translational Research Program, Yong Loo Lin School of Medicine, National University of Singapore, Singapore 117597, Singapore.; 10 Theranostics Center of Excellence (TCE), Yong Loo Lin School of Medicine, National University of Singapore, Singapore 138667, Singapore.; 11 Institute of Molecular and Cell Biology, Agency for Science, Technology, and Research (A*STAR), Singapore 138673, Singapore.

**Keywords:** ribozyme, chimeric permuted intron-exon system (CPIE), circular RNA (circRNA), mRNA therapeutics, size exclusion chromatography

## Abstract

**Rationale**: Circular RNA (circRNA) therapeutics hold great promise as an iteration strategy in messenger RNA (mRNA) therapeutics due to their inherent stability and durable protein translation capability. Nevertheless, the efficiency of RNA circularization remains a significant constraint, particularly in establishing large-scale manufacturing processes for producing highly purified circRNAs. Hence, it is imperative to develop a universal and more efficient RNA circularization system when considering synthetic circRNAs as therapeutic agents with prospective clinical applications.

**Methods:** We initially developed a chimeric RNA circularization system based on the original permuted intron-exon (PIE) and subsequently established a high-performance liquid chromatography (HPLC) method to obtain highly purified circRNAs. We then evaluated their translational ability and immunogenicity. The circRNAs expressing human papillomavirus (HPV) E7 peptide (43-62aa) and dimerized receptor binding domain (dRBD) from SARS-CoV-2 were encapsulated within lipid nanoparticles (LNPs) as vaccines, followed by an assessment of the *in vivo* efficacy through determination of antigen-specific T and B cell responses, respectively.

**Results:** We have successfully developed a universal chimeric permuted intron-exon system (CPIE) through engineering of group I self-splicing introns derived from Anabaena pre-tRNA^Leu^ or T4 phage thymidylate (Td) synthase gene. Within CPIE, we have effectively enhanced RNA circularization efficiency. By utilizing size exclusion chromatography, circRNAs were effectively separated, which exhibit low immunogenicity and sustained potent protein expression property. *In vivo* data demonstrate that the constructed circRNA vaccines can elicit robust immune activation (B cell and/or T cell responses) against tumor or SARS-CoV-2 and its variants in mouse models.

**Conclusions:** Overall, we provide an efficient and universal system to synthesize circRNA *in vitro*, which has extensive application prospect for circRNA therapeutics.

## Introduction

mRNA therapeutics represent a cutting-edge modality that leverages intrinsic cells as factories for protein synthesis, providing accelerated development timelines in contrast to traditional pharmaceuticals 1-3. In light of the COVID-19 pandemic, significant advancements have been made in mRNA-based vaccines, leading to a fervent pursuit of mRNA therapeutics, particularly in the fields of viral and tumor vaccines 4. However, there remain unresolved technical hurdles pertaining to stability, immunogenicity and translation efficiency that necessitate attention.

In comparison to linear mRNAs, circRNAs with closed loop structures have emerged as intriguing alternatives owing to their superior stability, sustained expression profile, and low immunogenicity 5-7. Due to these advantages, the number of circRNA based therapeutics under clinical development is growing rapidly 8. Nevertheless, the efficiency of RNA circularization remains a significant constraint, particularly in establishing large-scale manufacturing processes for producing highly purified circRNAs. Hence, it is imperative to dedicate more efforts towards achieving sufficient circularization efficiency.

*In vitro* synthesis of circRNAs typically involves the circularization of linear RNA fragments. Various methodologies have been developed for ligating linear RNA, encompassing chemical ligation, enzymatic ligation, and ribozyme-based approaches 9. Chemical ligation of 5' and 3' ends from a single-stranded RNA (ssRNA) to generate circRNA is accomplished by employing cyanogen bromide (BrCN) or 1-ethyl-3-(3'-dimethylaminopropyl) carbodiimide (EDC) 10. However, this approach poses certain challenges, including limited ligating efficiency and concerns regarding biosafety. Enzymatic ligation strategies employ DNA or RNA ligase derived from the bacteriophage T4 to catalyze ligation reactions 11. Despite the facilitation of RNA circularization through the utilization of DNA/RNA splint or meticulously designed internal secondary structures at the reaction ends, this approach is susceptible to significant intermolecular end-joining side reactions, thereby complicating the acquisition of pure circRNAs 10. Compared to chemical and enzymatic ligation methods, the utilization of self-splicing intron ribozymes in RNA circularization offers several advantages including enhanced circularization efficiency of linear RNA precursors, simplified reaction conditions, and purification methods 12-14.

Group I self-splicing introns possess a catalytic core capable of executing a series of transesterification reactions, resulting in the excision of the intron itself from the pre-mRNA and subsequent ligation of the adjacent exons 9. The PIE system involves engineered RNA sequences with rearranged intronic and exonic regions, thereby generating an artificial sequence where conventional exon sequences are positioned within the intronic regions 15, 16. In 2018, Wesselhoeft *et al.* developed a highly efficient engineered PIE system (referred to as ePIE) in which the splicing bubble is firmly locked to ensure the efficient circularization of diverse RNA sequences through rational design of external and internal hybridization arms 17. Despite these advancements, certain concerns persist within this system. For instance, the inclusion of long scar sequences derived from exons of Anabaena pre-tRNA^Leu^ or T4 phage Td synthase gene and the formation of double-stranded RNA (dsRNA) structures *via* internal hybridization arm may potentially trigger an innate immune response in host cells 18. Although attempts have been made in other studies to obtain scarless circRNAs and avoid their immunogenicity, these strategies rely on the secondary structure or sequence of linear precursor RNA, which limits their versatility to some extent 11, 19.

In the current study, we designed an enhanced CPIE system based on group I self-splicing introns. The CPIE system strategically arranges two group I introns along with the desired sequence to achieve efficient circularization of RNA molecules. Remarkably, the CPIE system not only enhances the efficiency of RNA circularization but also minimizes the presence of scar sequences in the final circRNA products, thereby mitigating potential immunogenicity concerns. Serving as an innovative engineering platform for *in vitro* circRNA preparation, the CPIE system successfully facilitated the production of circRNA vaccines targeting both tumor and infectious diseases, highlighting its immense potential for advancing circRNA therapeutics.

## Results

### Design of the CPIE system

The ribozyme method utilizing permuted group I catalytic intron stands out as a preferable approach for RNA circularization *in vitro*, requiring only the addition of GTP and Mg^2+^ as cofactors 17, 20, 21. In our initial investigations, we utilized previously developed PIE systems, known as T4 nPIE and Ana nPIE, employing native intron sequence from T4 phage Td synthase and Anabaena pre-tRNA^Leu^ genes, respectively, to produce the circRNA. However, upon sequential insertion of the internal ribosome entry site (IRES) from Coxsackievirus B3 (CVB3) and an optimized enhanced green fluorescent protein (EGFP)-coding sequence into both the T4 nPIE and Ana nPIE systems (Fig. S1), we did not observe notable precursor RNA circularization within a 30-min timeframe, which is consistent with previous findings in other studies 17.

**Figure 1:**
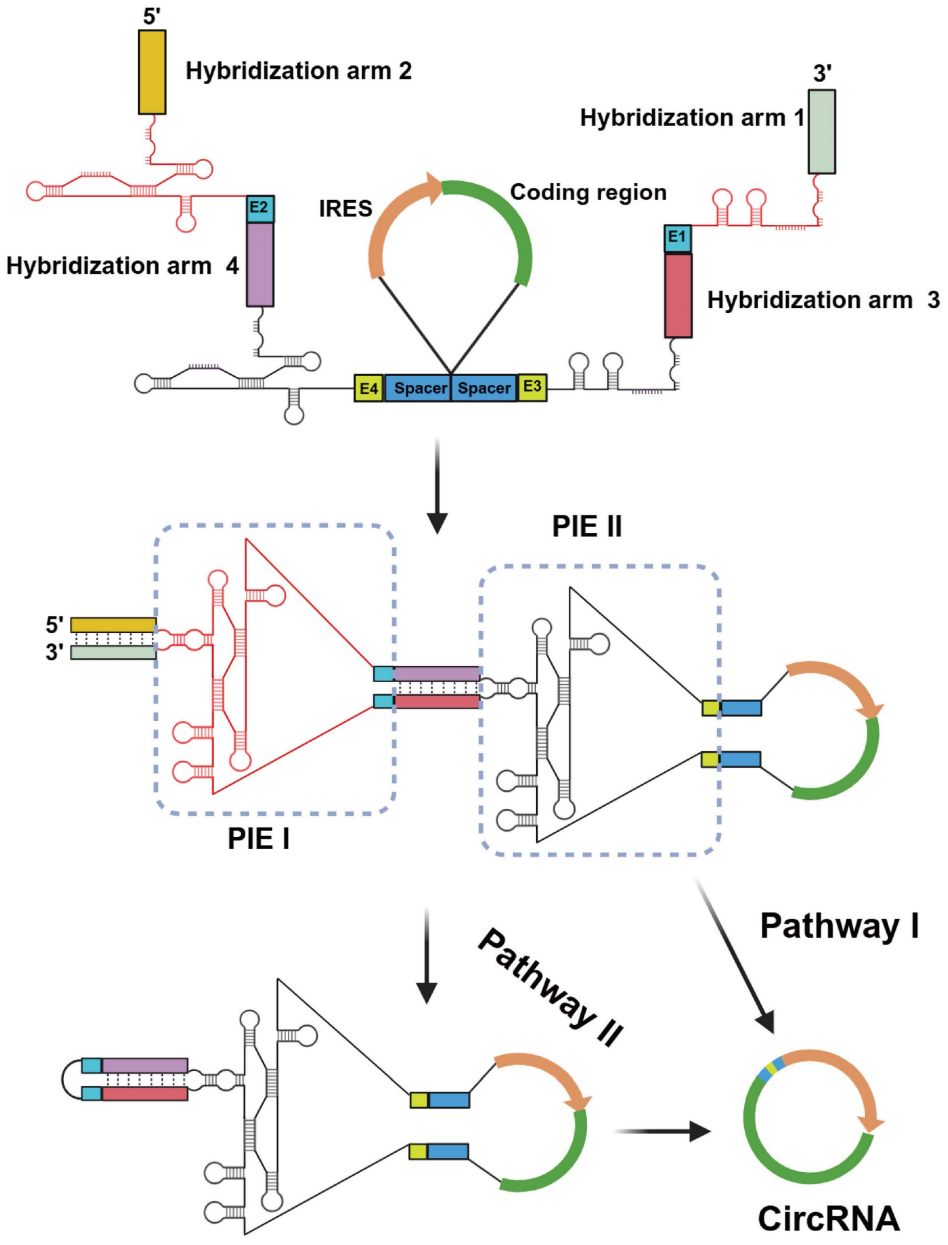
** Design of the CPIE system for circRNA synthesis.** A schematic diagram depicts the components of the CPIE system, along with its hypothetical splicing pathways. In pathway I, the PIE I module can facilitate the folding process of the PIE II module and expedite circularization. In pathway II, the PIE I module initially generates a circular intermediate RNA, followed by an intramolecular splicing reaction mediated by the PIE II module. The splicing sites (E1-E4), spacers and hybridization arms of the CPIE system are indicated.

In recent studies, the utilization of a longer hybridization arm has been frequently employed to enhance the performance of PIE 11, 19. However, it is important to note that longer does not necessarily equate to better results. In fact, in certain cases, these auxiliary sequences may also have an adverse impact on RNA transcription or splicing reactions 11, 19. To achieve the high-efficiency RNA circularization with correct splicing, we designed an engineered CPIE system through strategically arranging two highly structured group I introns from T4 phage and Anabaena (Fig. 1). In our system, the linear precursor RNA can produce circRNA through two pathways. On the one hand, with the assistance of cofactors, the precursor RNA can directly produce circRNA *via* internal PIE II module. Under this circumstance, PIE I module can quickly assist the PIE II module in folding *via* nucleotide interaction. On the other hand, the precursor RNA first produces a circular intermediate RNA *via* external PIE I module and then intramolecular splicing reaction occurs *via* internal PIE II module to produce the final circRNAs (Fig. 1).

### CPIE system facilitates RNA circularization

**Figure 2:**
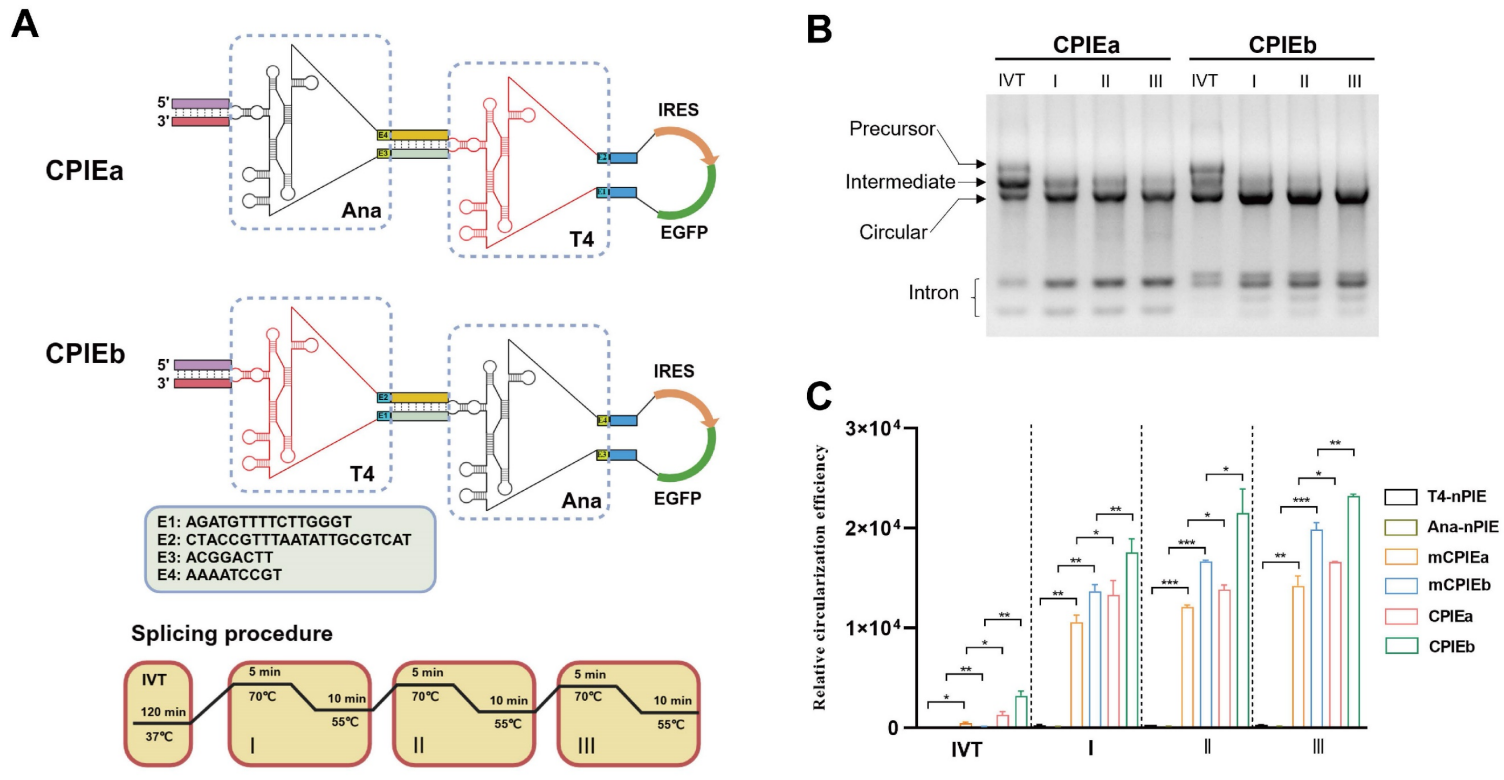
** Characterization of the CPIE system.** (A) Schematic diagrams illustrating the engineered CPIEa and CPIEb systems and splicing procedure are presented. (B) Precursor RNA circularization within CPIEa and CPIEb systems are evaluated, separately. The bands corresponding to putative precursor RNA, intermediate RNA, circRNA, and free intron are indicated. (C) The efficiency of RNA circularization using the CPIEa and CPIEb systems is quantified by RT-qPCR. Data are shown as mean ± sd. Statistical analysis was carried out by using unpaired two-tailed *t*-tests (*P < 0.05, **P < 0.01, ***P < 0.001).

To validate the design concept of CPIE, two distinct CPIE systems, namely CPIEa and CPIEb, were developed. In the case of CPIEa, the PIE module of Anabaena intron is positioned externally, while the PIE module of T4 phage intron is located internally. Conversely, in CPIEb, these PIE modules are arranged in a reversed manner (Fig. 2A). Similarly, the CVB3 IRES and EGFP-coding sequence were inserted into the spacer-spacer junctions of both CPIEa and CPIEb systems. After undergoing *in vitro* transcription (IVT) and splicing procedures, circRNAs were noticeably generated (Fig. 2B).

To further ascertain the functionality of designed components in CPIE system, we introduced mutations to the splicing sites of the PIE I module within CPIEa and CPIEb systems, resulting in mCPIEa and mCPIEb respectively (Fig. S1). These mutated variants, along with T4-nPIE and Ana-nPIE, were employed as controls in our assays of RNA circularization. The RNA circularization efficiency in their RNA products obtained from IVT and splicing reaction was evaluated using quantitative reverse transcription PCR (RT-qPCR). Compared to T4-nPIE and Ana-nPIE, mCPIEa and mCPIEb exhibited higher circularization efficiency, indicating the significance of the PIE I module even in an inactive state for RNA circularization. Moreover, both CPIEa and CPIEb systems exhibited superior circularization efficiency compared to the mCPIEa and mCPIEb system (Fig. 2C). This phenomenon has also been observed during IVT (Fig. 2C), further suggesting that a catalytically active PIE I module would enhance PIE II-mediated RNA circularization.

### Characterization of circRNA prepared with CPIE systems

**Figure 3:**
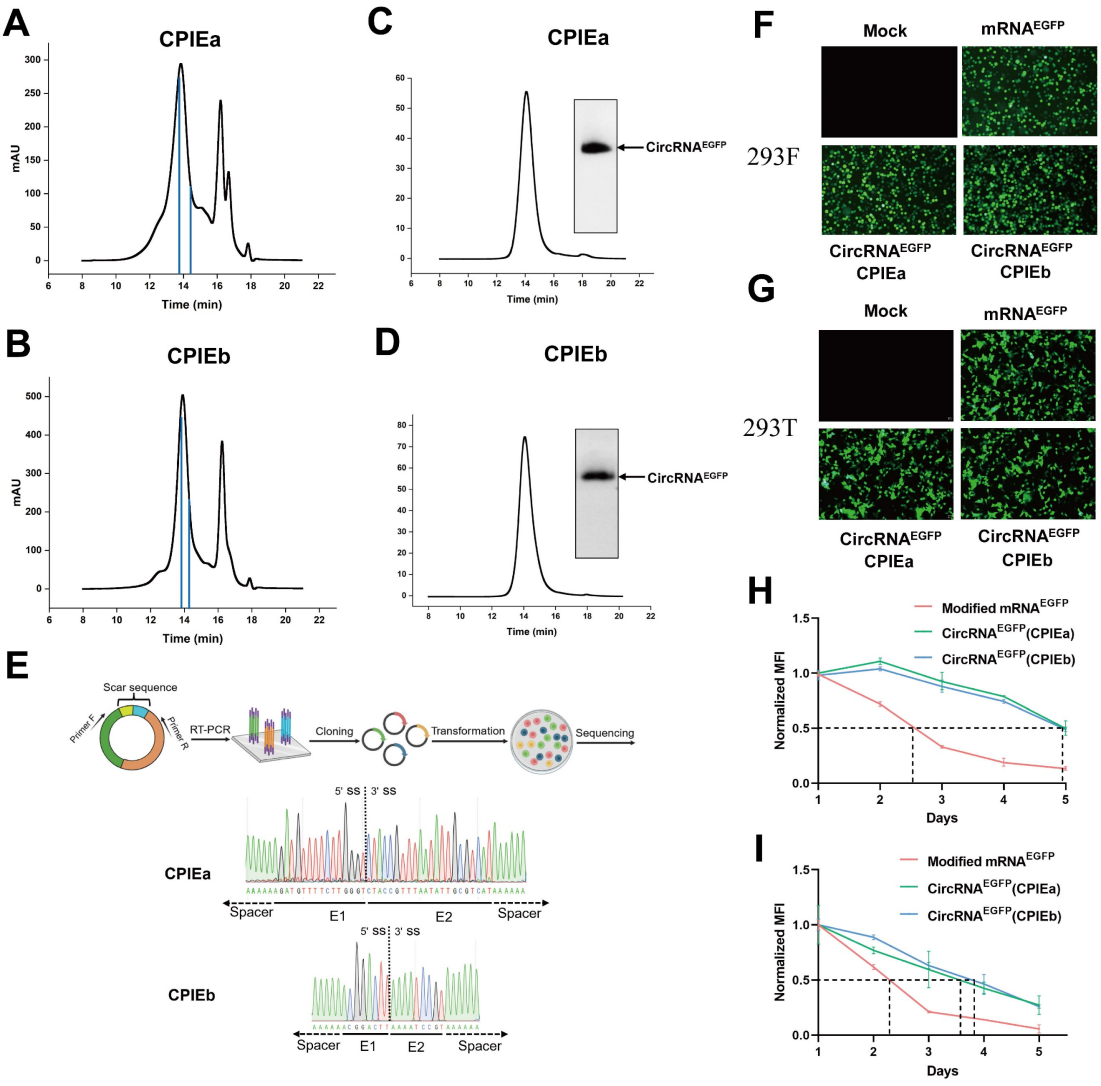
**The purification and expression of circRNA.** (A-B) HPLC chromatogram analysis was performed on the final circRNA products generated using CPIEa (A) and CPIEb (B) systems, respectively. The retention time corresponding to the collection of samples has been indicated. (C-D) Agarose gel electrophoresis results were obtained along with HPLC chromatograms of collected circRNA^EGFP^ produced through CPIEa (C) and CPIEb (D) systems, respectively. (E) RT-PCR was performed to target the putative splice junction, followed by Sanger sequencing for validation of the splicing sites of circRNAs generated by the CPIEa and CPIEb systems. A representative sequencing analysis is provided. (F-G) Fluorescence microscopy imaging was conducted at 24 h after transfecting HEK293F (F) and HEK293T (G) cells with circRNA^EGFP^ and m1ψ-modified linear mRNA expressing EGFP. (H-I) The mean fluorescence intensity (MFI) change in the HEK293F (H) and HEK293T (I) cells was measured starting from 24 h after transfection with either circRNA^EGFP^ or m1ψ-modified linear mRNA expressing EGFP, and this measurement continued for 5 days. All MFI data were normalized with their respective MFI values at 24 h.

To obtain highly purified circRNA, we employed the HPLC method to isolate circRNA from the circularized mixture generated through CPIEa and CPIEb systems (Fig. 3A, B). Subsequently, agarose gel electrophoresis and HPLC procedures were performed to validate the purity of the circRNA by confirming effective elimination of impurities such as precursor RNA and free introns (Fig. 3C, D). The purified circRNA was subsequently subjected to reverse transcription-PCR (RT-PCR) and Sanger sequencing for confirmation of the splicing sites in the CPIE system, all of which exhibited accurate ligation of exons as expected (Fig. 3E).

For the translational evaluation of circRNA in cellular systems, HPLC-purified circRNA^EGFP^ derived from CPIEa and CPIEb methodologies, along with m1ψ-modified linear mRNA, were transfected into HEK293F and HEK293T cells. Subsequently, the fluorescence intensities were continuously monitored over a 5-day period. The circRNA prepared using CPIEa and CPIEb exhibited comparable levels of EGFP expression to that of m1ψ-modified linear RNA (Fig. 3F and 3G). However, circRNA demonstrated prolonged protein expression compared to that of linear mRNA (Fig. 3H, 3I, Fig. S2 and S3). Specifically, in HEK293F cells, the protein expression half-lives of EGFP from CPIEa and CPIEb were approximately 118 h, compared to around 60 h for modified linear mRNA. In HEK293T cells, the protein expression half-lives of EGFP from CPIEa and CPIEb were roughly 90 h, compared to about 55 h for modified linear mRNA (Fig. 3G and 3I). These findings support the previously reported enhanced stability and enduring expression of circRNA.

### CircRNA prepared with CPIE system exhibits low immunogenicity

In addition to considering circularization efficiency, it is imperative to consider the immunogenicity of *in vitro* synthesized circRNA when exploring its potential applications. Recent studies have highlighted that the recognition of *in vitro* synthesized circRNA by immune sensors may be attributed to the presence of residual scar sequence, thereby triggering an innate immune response 18, 22, 23. Conversely, some researchers argue that *in vitro* synthesis of circRNA does not elicit an immunogenic response and propose that any cellular immune reactions are attributed to the presence of a small amount of linear RNA contaminants within the circRNA products. This viewpoint is supported by successful elimination of unwanted cellular immune responses through highly purified circRNA samples 7, 13, 24, 25. Our CPIEa and CPIEb systems only keep minimal scar sequences (CPIEa, 38 bp; CPIEb, 17 bp) for splicing in final circRNA products, which is significantly lower than previous ePIE (108 bp) system (Fig. 2A and 3E).

**Figure 4:**
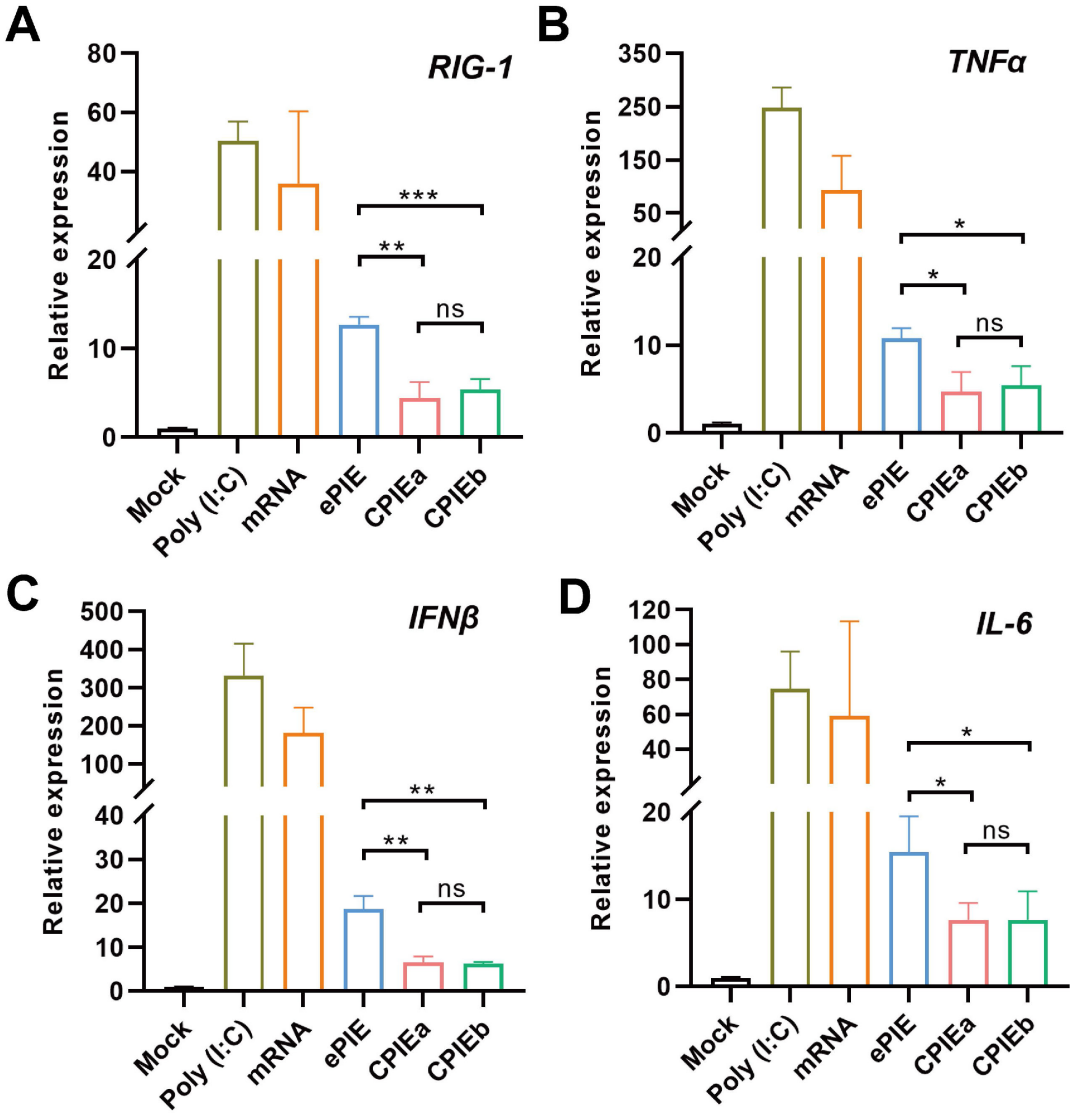
** Immunogenicity of the circRNA generated by CPIE systems.** RT-qPCR analysis was performed to evaluate the relative expression of specific genes,* RIG-1* (A), *TNFα* (B), *IFNβ* (C) and *IL-6* (D) in A549 cells transfected with ePIE- and CPIE-generated circRNA^EGFP^ at 12 h post-transfection. The mRNA expression levels were normalized using the reference gene *actin*, and compared to those observed in mock-transfected cells. Notably, Poly(I:C) and unmodified linear mRNA^EGFP^ served as controls for this study. Data are shown as mean ± sd. Statistical analysis was carried out by using unpaired two-tailed *t*-tests (ns: not significant, *P < 0.05, **P < 0.01, ***P < 0.001).

Following HPLC purification, we compared the innate immune responses of circRNA^EGFP^ prepared with CPIE and ePIE systems through RT-qPCR analysis employing specific primers targeting *RIG-1* as well as genes encoding inflammatory cytokines such as *TNFα*, *IFNβ*, and *IL-6* in A549 cells. As a result, both circRNAs prepared using CPIE and ePIE exhibited reduced innate immune responses in A549 cells compared to unmodified linear mRNA^EGFP^ after a 12-hour transfection period. However, the circRNA prepared using CPIE induced more pronounced attenuation of innate immune responses than the ePIE system (Fig. 4A-D). Furthermore, in consideration of the potential impact of transfection efficiencies and RNA stability on their immunogenicity, we additionally utilized intracellular mRNA^EGFP^ or circRNA^EGFP^ quantities as normalization factors for the expression levels of *RIG-1*, *TNFα*,* IFNβ*, and *IL-6*. The results also demonstrated that circRNA exhibited reduced immunogenicity compared to unmodified linear mRNA, and circRNA prepared via CPIE induced lower immunogenicity than that obtained through ePIE (Fig. S4). Collectively, these findings highlight the reduced potential immunogenicity associated with circRNA generated using the CPIE system.

### Preparation of LNP@circRNA complex

**Figure 5:**
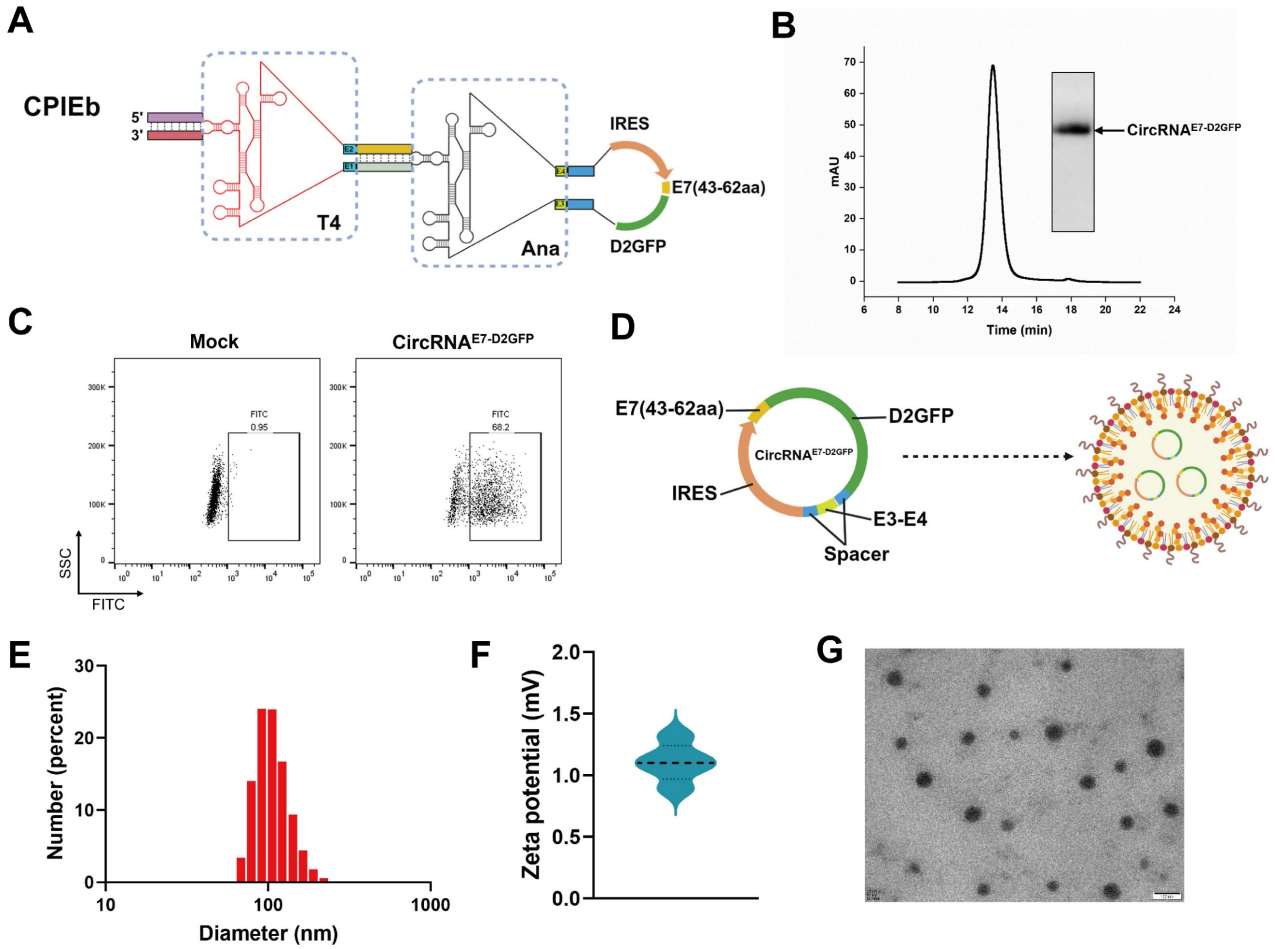
** Preparation of the LNP@circRNA^E7-D2GFP^ complex.** (A) Schematic representation illustrating the components of the IVT template for circRNA^E7-D2GFP^ preparation. (B) Agarose gel electrophoresis result and HPLC chromatogram depicting the collected circRNA^E7-D2GFP^ generated through CPIEb system. (C) The expression of circRNA^E7-D2GFP^ was assessed in HEK293F cells using flow cytometry after transfection for 24 h. (D) Schematic depiction of the LNP@circRNA^E7-D2GFP^ complex formation. (E) Size distributions analysis conducted on the LNP@circRNA^E7-D2GFP^ complex. (F) Zeta potential evaluation performed on the LNP@circRNA^E7-D2GFP^ complex. (G) TEM image displaying the morphology of the LNP@circRNA^E7-D2GFP^ complex. Scale bar, 100 nm.

Given the evident advantage of the CPIEb system in RNA circularization, we endeavored to generate circRNA for evaluating its potential application in circRNA therapeutics. Initially, we substituted the EGFP-coding sequence of the CPIEb IVT template with a fusion construct comprising a destabilized green fluorescent protein (D2GFP) reporter and an antigen derived from HPV E7 (43-62aa) (Fig. 5A). After the IVT and splicing reactions were performed, purification of circRNA^E7-D2GFP^ was carried out using the HPLC method (Fig. 5B). Subsequently, transfection of circRNA^E7-D2GFP^ into HEK293F cells was conducted to assess its protein translation. The expression of E7-D2GFP was validated through flow cytometry analysis (Fig. 5C). To achieve efficient *in vivo* delivery, encapsulation of circRNA^E7-D2GFP^ with LNP resulted in the formation of a stable complex (Fig. 5D). The average diameter of LNP@circRNA^E7-D2GFP^ complex was determined to be approximately 100 nm using dynamic light scattering (DLS) measurements (Fig. 5E), while the corresponding zeta potential was measured to be around 1.1 mV (Fig. 5F). Additionally, characterization *via* transmission electron microscopy (TEM) confirmed the morphology of circRNA^E7-D2GFP^ as spherical nanoparticles (Fig. 5G).

### CircRNA^E7-D2GFP^ vaccine induces potent anti-tumor immunity

**Figure 6:**
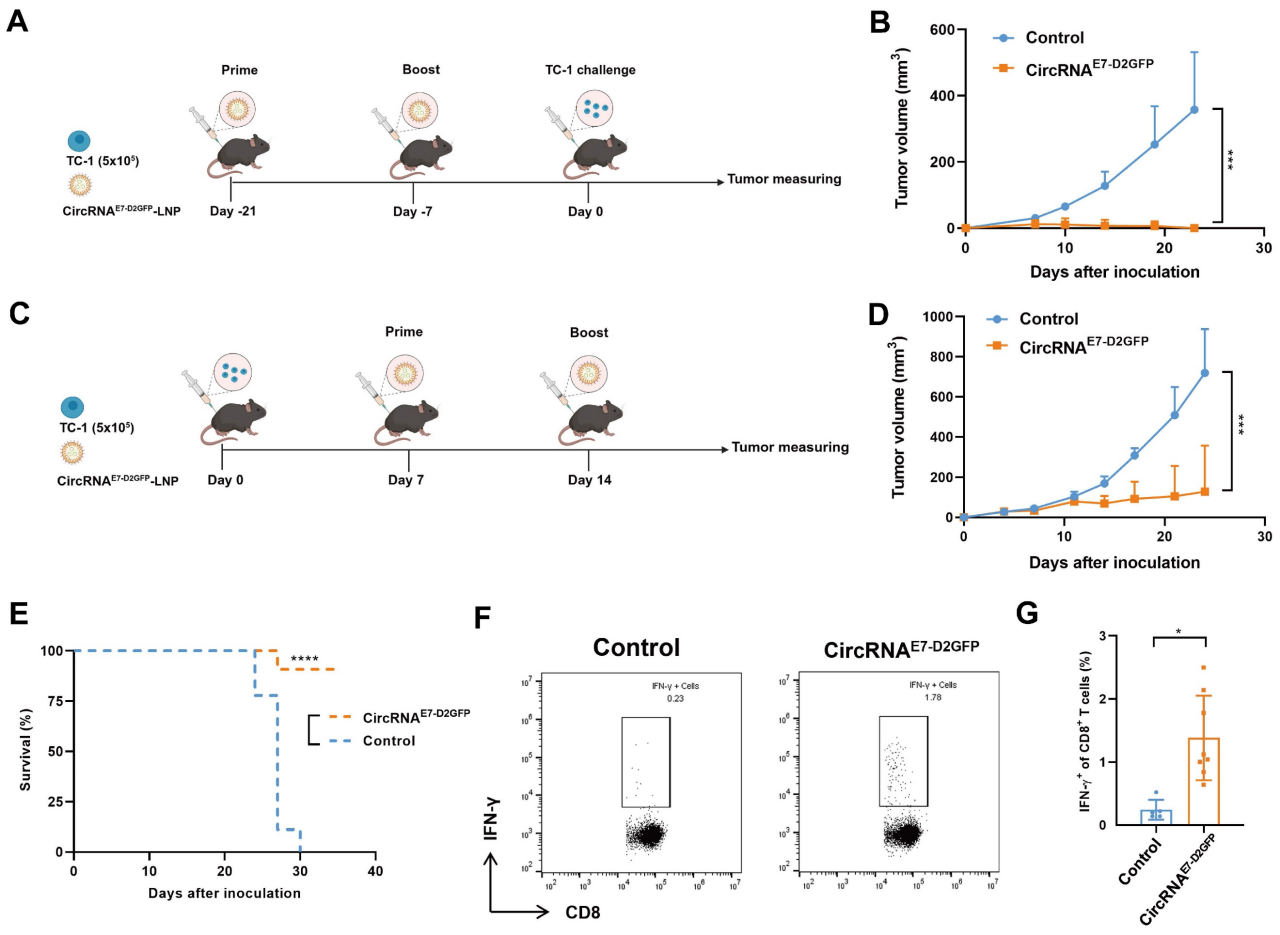
** LNP@CircRNA^E7-D2GFP^ induces robust T cell responses and demonstrates efficacy as both prophylactic and therapeutic anti-tumor vaccines.** (A) Timeline of the anti-tumor assay using LNP@circRNA^E7-D2GFP^ as a prophylactic vaccine in TC-1 tumor model is presented. (B) C57BL/6J mice (n = 5-6), which were immunized twice with circRNA^E7-D2GFP^ or vehicle control, were challenged with TC-1 cells (5 × 10^5^). (C) Timeline of the anti-tumor assay using LNP@circRNA^E7-D2GFP^ as a therapeutic vaccine in TC-1 tumor model is depicted. (D-E) TC-1 tumor-bearing C57BL/6J mice (n = 9) were treated twice with circRNA^E7-D2GFP^ or vehicle control as indicated. Tumor growth (D) and survival curves (E) are shown. (F-G) Seven days after second immunization, lymphocytes from the spleens were isolated and stimulated with E7 peptide (49-57aa). IFN-γ^+^CD8^+^ T cells were analyzed by flow cytometry, representative results (F) and quantification (G) of the data were shown. Data are shown as mean ± sd. *P* value was determined by log-rank test (E) or unpaired two-tailed *t*-tests (*P < 0.05, **P < 0.01, ***P < 0.001) (B, D and G).

To evaluate the potential of circRNA^E7-D2GFP^ as a tumor vaccine, we initially administered two immunizations of LNP@circRNA^E7-D2GFP^ containing 10 µg circRNA to C57BL/6J mice at 2-week intervals. Subsequently, TC-1 challenge was conducted to assess the efficacy of the circRNA^E7-D2GFP^ vaccine (Fig. 6A). We found that all mice were tumor free in the vaccinated group, whereas the control group exhibited significant tumor progression, suggesting that circRNA^E7-D2GFP^ serves as an efficacious prophylactic vaccine against HPV-related tumors (Fig. 6B and Fig. S5A). We also evaluated the therapeutic potential of circRNA^E7-D2GFP^. TC-1 tumor-bearing mice were immunized twice at a 7-day interval (Fig. 6C). Our results demonstrate that circRNA^E7-D2GFP^ vaccine could effectively trigger E7-specific T cell responses, suppress tumor progression, and significantly prolong the survival (Fig. 6D-G, Fig. S5B and S6). These findings highlight the promising application of CPIEb-generated circRNAs as both prophylactic and therapeutic platforms for anti-tumor vaccines.

### CircRNA^dRBD^ vaccine elicits broad immune responses against SARS-CoV-2 and its variants

**Figure 7:**
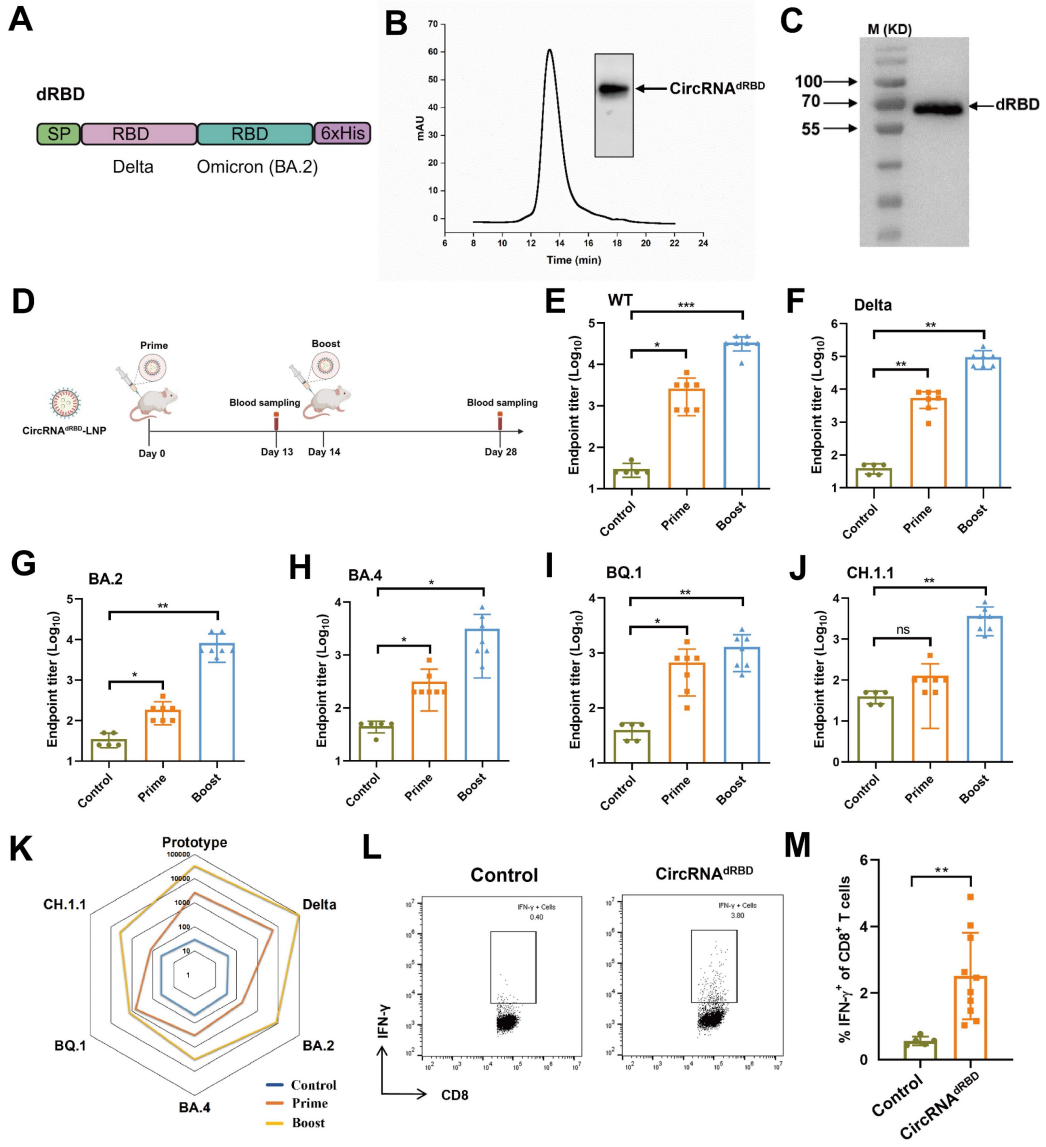
** CircRNA^dRBD^ vaccine induces potent B cell and T cell immune responses against SARS-CoV-2.** (A) Schematic representation of the dRBD antigen is depicted. SP: signal peptide. (B) The agarose gel electrophoresis result and HPLC chromatogram illustrate the collected circRNA^E7-D2GFP^ generated through CPIEb system. (C) The Western blot result demonstrates the expression of circRNA^dRBD^. (D) A timeline outlining the immunization and assessment process to evaluate the circRNA^dRBD^ vaccine is provided. BALB/c mice (n = 5-7) were immunized with circRNA^dRBD^ or vehicle control at a 2-week interval. Serum samples were collected 13 days after the first immunization and 14 days after the second immunization. (E-J) ELISA assays reveal specific total IgG titers against RBD from SARS-CoV-2 (E), as well as its delta (F), BA.2 (G), BA.4 (H), BQ.1 (I), and CH.1.1 (J) variants. (K) A radar map comparison showcases specific IgG titers against RBD of SARS-CoV-2 and its variants. (L-M) Seven days after second immunization, lymphocytes from the spleens were isolated and stimulated with dRBD fusion protein. IFN-γ^+^CD8^+^ T cells were analyzed by flow cytometry, representative results (l) and quantification (m) of the data were shown. Data are shown as mean ± sd. Statistical analysis was carried out by using unpaired two-tailed *t*-tests (ns: not significant, *P < 0.05, **P < 0.01, ***P < 0.001).

We utilized the engineered CPIEb system to generate circRNA that encodes an antigen comprising tandem RBD domains derived from the delta and omicron variants (BA.2) of SARS-CoV-2, referred to as circRNA^dRBD^ (Fig. 7A). Following HPLC purification (Fig. 7B), the circRNA^dRBD^ was transfected into HEK293F cells. Western blot analysis confirmed successful expression of the recombinant dRBD antigen (Fig. 7C). To evaluate its potential as a vaccine candidate, BALB/c mice were immunized with LNP@circRNA^dRBD^ containing 10 µg circRNA at 2 weeks interval. Serum samples were collected 13 days after the initial immunization and 14 days after the second immunization as indicated in figure 7D. The enzyme-linked immunosorbent assay (ELISA) demonstrated that the circRNA^dRBD^ vaccine elicited a robust and broad antibody response against SARS-CoV-2, including its various variants such as delta, BA.2, BA.4, BQ.1, and CH.1.1 (Fig. 7E-J). Notably, this response was particularly potent against the prototype strain of SARS-CoV-2 as well as the delta, and omicron BA.2 variants (Fig. 7K). Additionally, the circRNA^dRBD^ vaccine effectively activated RBD-specific CD8^+^ T cells (Fig. 7L, M and Fig. S6), indicating its ability to induce both humoral and cellular immune responses against SARS-CoV-2.

## Discussion

CircRNA was discovered in 1976 26, and was initially considered as a byproduct of cellular mRNA splicing errors. However, over the past decade, numerous studies have highlighted the functional role of circRNA within cells, elevating circRNAs to the status of pivotal molecules involved in cell differentiation, tissue homeostasis, disease progression, and immune regulation 27-29. The discovery of circRNA's ability to express proteins has provided a novel perspective on intracellular protein synthesis, beyond the traditional mRNA translation 30-33. In 2018, Wesselhoeft *et al.* demonstrated that engineered circRNA can effectively and durably express proteins in eukaryotic cells, thereby opening new avenues for exogenous circRNA applications 17. Furthermore, their findings suggest that circRNA may serve as an effective alternative to linear mRNA in certain cases due to its superior stability, sustained expression profile, and limited immunogenicity 7, 17.

The ribozyme strategy is the preferred choice for catalyzing the circularization of linear RNA substrates in circRNA *in vitro* synthesis due to its relatively mild reaction conditions and simplified purification methods. However, large-scale circRNA synthesis and downstream application still encounter numerous challenges. In the context of the PIE system, Wesselhoeft *et al.* employed both external and internal hybridization arms to facilitate circularization 17. Nevertheless, the elongation of the external hybridization arm leads to competition with other core regions of the ribozyme, resulting in incorrect splicing and/or limited efficacy in circularization 13. Additionally, incorporating a longer internal hybridization arm may trigger an innate immune response due to the formation of dsRNA structure by the hybridization arm in final circRNA products 18.

Within the CPIE system, two independent splicing bubbles are fixed and isolated through medium-length (20 bp) hybridization arms. Despite the presence of chimeric internal and external group I elements that contribute to an increase in both length and complexity of the PIE structure, these elements are confined within their respective structures and synergistically complement each other without engaging in competition with other regions. Consequently, the CPIE system demonstrates exceptional efficiency in circularization. In CPIE, the external PIE can replace the hybridization arm in the previous system to improve the efficiency of internal PIE-mediated RNA cyclization. In addition, external PIE enhances the efficiency of circular intermediate formation, which enables the internal PIE to initiate reactions within the molecule, thereby facilitating the splicing process and significantly increasing the overall circulation efficacy. Our data unequivocally demonstrate an impressive final circulation efficiency even without employing internal hybridization arms.

The immunogenicity of circRNA prepared using the ribozyme method *in vitro* has been a subject of controversy. Understanding the immunogenicity of circRNA is crucial, particularly in therapeutic applications, to ensure safety and predict potential immune responses. Generally, circRNAs are believed to exhibit lower immunogenicity compared to linear mRNAs due to their closed-loop structure, which may reduce recognition by immune sensors 7. Recent studies have indicated that extraneous fragments such as exonic scars in circRNA prepared *via* the PIE method pose a potential risk for unwanted innate immune responses 18, 19. Therefore, the goal of newly developed ribozyme-based circRNA preparation platforms is to reduce or eliminate scar sequences. As a versatile RNA circularization platform, ePIE generates a long (108 bp) exon-exon junction that is retained as a scar in the final circRNA products 17. However, despite the development of novel approaches such as Clean-PIE and CirCode strategies to produce scarless circRNA by concealing the splicing site within either ORF or IRES sequences, their versatility is hindered due to their strong dependence on RNA sequence and structure 14, 19. In this study, we have endeavored to simplify the exon sequences of our designed CPIE system to the greatest extent possible. As a result, we have successfully generated a negligible scar sequence (CPIEa, 38 bp; CPIEb, 17 bp) in final circRNA products by employing the CPIE system. In summary, our innovative approach effectively addresses potential innate immune responses associated with prepared circRNA while also ensuring its universality in various applications.

Another prevailing perspective suggests that higher purity of circRNA is associated with lower immunogenicity. Some investigators even argue that residual linear RNA and compact introns in the structure are the primary risk factors for circRNA's immunogenicity 7. Despite the establishment of several purification methods, such as HPLC and column purification, the degree of separation appears to be insufficient 17, 34. In this study, we meticulously collected the circRNA products corresponding to the chromatogram using HPLC method. Upon transfection into A549 cells, we observed a reduced immune response elicited by CPIE-generated circRNA compared to ePIE-generated circRNA. This implies that reducing the sacr sequence is advantageous in decreasing the immunogenicity of circRNA. However, irrespective of whether circNRA is generated by CPIE or ePIE, they still elicited a discernible immune response compared to the mock group, indicating that the current methodologies employed for circRNA preparation are unable to completely eliminate its immunogenicity. The immunogenicity of circRNA may vary across different cell types, tissues, or physiological contexts, thereby impacting their interaction with the immune system. Therefore, investigating the immunogenicity in diverse cellular environments will further enhance our understanding of circRNA-mediated immune responses. In addition, the evaluation of circRNA immunogenicity remains a formidable task due to the necessity for highly sensitive detection techniques and standardized characterization of immune responses triggered by diverse circRNA species. Consequently, more comprehensive investigations are warranted to unravel the specific determinants that influence circRNA immunogenicity and their implications in various biological contexts, thereby advancing our understanding of this emerging field.

Vaccines are the most extensively investigated and clinically accepted application of mRNA-based therapeutics 35, 36. Although circRNA vaccines have not yet been utilized in clinical settings, several studies, including our own data, have demonstrated that circRNA vaccines exhibit enhanced levels of antigen expression and prolonged antigen persistence compared to mRNA vaccines 17, 20. This leads to the generation of high titers of total and neutralizing antibodies 37. Furthermore, in certain cases, the immunogenicity of circRNA as vaccines without adjuvants may offer potential benefits for improving immune responses. The circRNA generated by the CPIE system, as mentioned previously, still exhibits a relatively weaker immunogenicity, which may potentially confer an advantage for our vaccines targeting tumors and SARS-CoV-2. In addition, there is a rapid expansion in the field of circRNA-based therapeutics, encompassing diverse approaches such as protein replacement, gene editing, and the generation of CAR-T/TCR-T cells in situ 38-42. The utilization of an immune evasion circRNA in these fields is preferred due to its potential for mitigating adverse effects and treatment risks. On one hand, it is imperative to develop innovative methodologies for *in vitro* preparation of immune evasion circRNA. On the other hand, the development of novel targeted delivery approaches to circumvent immunological recognition of circRNA may also be a pivotal strategy.

## Materials and methods

### Design and preparation of IVT templates

For IVT templates for circRNA synthesis, the T7 RNA polymerase promoter (TAATACGACTCACTATAGGG), engineered group I self-splicing intron, and CVB3 IRES sequences were chemically synthesized (Logenbio, China) and cloned into a commercially available pUC57 plasmid. Protein coding sequences such as EGFP, E7-D2GFP, and dRBD were obtained from lab-preserved plasmids and inserted into the synthetic plasmid using the Gibson assembly method based on experimental requirements. The IVT template for linear mRNA synthesis was prepared according to the Takara IVTpro^TM^ mRNA Synthesis System (Takara, Japan). Table S1 contains all the sequences for IVT templates. *Escherichia coli* DH5α cells were cultured in LB medium at 37°C for plasmid construction purposes.

### Linear mRNA and circRNA synthesis

The plasmids were constructed and subsequently linearized using a designated restriction enzyme, followed by extraction with a phenol/chloroform/isoamylol (25:24:1) solution. The linear unmodified mRNA was synthesized using EasyCap T7 Co-transcription Kit with CAG Trimer (Vazyme, China) as per manufacturer's instructions. The linear m1ψ-modified mRNA was synthesized using the same method with the complete replacement of uridine with N1-methylpseudouridine. After the DNA template was removed by DNase I (Takara, Japan) at 37°C for 30 minutes, the linear mRNA products were column-purified using a HiPure RNA Pure Micro kit (Magen, China). For circRNA synthesis, the linearized plasmid DNA was utilized as the template for IVT *via* the T7 High Yield RNA Synthesis Kit (New England Biolabs, USA). The resulting IVT products were treated with DNase I (Takara, Japan) at 37°C for 30 minutes before additional GTP was added to achieve a final concentration of 2 mM, along with a buffer containing magnesium (50 mM Tris-HCl, 10 mM MgCl_2_, 1 mM DTT, pH 7.5). Splicing reactions were performed according to our designed procedures outlined in Figure 2A. Specifically, the precursor RNA was subjected to a heat treatment at 70℃ for 5 minutes, followed by an immediate transfer to 55℃ for 10 minutes. This process was repeated three times. Subsequently, the circRNA was column-purified. The resulting column-purified circRNA samples were separated on 2% agarose gels.

### CircRNA purification and splice junction sequencing

To eliminate circRNA impurities, the HPLC assay was conducted using a 4.6×300 mm size exclusion column with a particle size of 5 µm and pore size of 1000 Å (Sepax Technologies, USA) on an Elite EClassical 3200 Series HPLC system (Elite, China). RNA samples were run in RNase-free phosphate buffer (150 mM sodium phosphate, pH 7.0) at a flow rate of 0.6 mL/minute. Detection and collection of RNA were performed through UV absorbance at 260 nm. The obtained RNA fractions underwent desalting through a desalting column, precipitation with ammonium acetate, and subsequent resuspension in RNase-free water. For splice junction sequencing analysis, reverse transcription was performed using the EasyScript One-Step gDNA Removal and cDNA Synthesis SuperMix (TransGen, China) to synthesize cDNA. A specific primer pair targeting the splice junction of putative circRNA was utilized in a standard PCR procedure to amplify PCR products from the synthetic cDNA template. These PCR products were then cloned into the pUC57 vector for subsequent sequencing analysis.

### Mice and cell lines

Female C57BL/6J or BALB/c mice, aged six to eight weeks, were purchased from Beijing Vital River Laboratory Animal Technology Co., Ltd. All mice were housed under specific pathogen-free conditions in the animal care facilities at the Tsinghua University. All animal experiments strictly complied with the standards (24-YGC1) of the Institutional Animal Care and Use Committee of Tsinghua University. HEK293F cells were cultured in SMM 293-TII medium (Sino Biological, China) and maintained in an incubator shaker at 37°C with 5% CO_2_. HEK293T cells were cultured in DMEM medium (Gibco, USA) with 10% fetal bovine serum and maintained in an incubator shaker at 37°C with 5% CO_2_. TC-1, a tumor cell line transformed from C57BL/6J primary mouse lung epithelial cells and expressing HPV16 E6 and E7 oncoproteins, was kindly provided by Dr. Xuemei Xu (Institute of Basic Medical Sciences Chinese Academy of Medical Sciences & School of Basic Medicine, Peking Union Medical College). A549 cells were adenocarcinomic human alveolar basal epithelial cells, purchased from the American Type Culture Collection. Both A549 and TC-1 cells were maintained in Dulbecco's modified Eagle's medium supplemented with 10% heat-inactivated fetal bovine serum, 100 U/mL penicillin, and 100 µg/mL streptomycin at 37 °C with 5% CO_2_.

### RNA transfection

For expression analysis, 2 µg of HPLC-purified circRNAs or an equimolar amount of m1ψ-modified mRNA were transfected into 4×10^6^ HEK293F cells/2 mL or 2×10^6^ HEK293T cells/2 mL per well of a 6-well plate using the polyethylenimine (PEI)-mediated method. For transfection in HEK293T cells, the serum-free DMEM medium was replaced with fresh DMEM medium containing 10% fetal bovine serum after a transfection period of 6 h. After culturing for 24 h, circRNA and mRNA expression were detected using appropriate techniques such as fluorescence microscopy, flow cytometry, or western blotting. Notably, the expressions of circRNA^EGFP^ and mRNA^EGFP^ were continuously monitored for a duration of 5 days to evaluate their persistence.

### Quantitative reverse transcription PCR (RT-qPCR)

A549 cells were transfected with 2 µg of HPLC-purified circRNAs or an equimolar amount of unmodified linear mRNA. The poly(I:C) used as a positive control. After 12 h of incubation, the cells were harvested and total RNA was extracted using the TransZol Up kit (TransGen, China). Subsequently, cDNA synthesis was performed using the EasyScript One-Step gDNA Removal and cDNA Synthesis SuperMix (TransGen, China). Quantitative PCR analysis was conducted on an Applied Biosystems 7500 system (Thermo Fisher Scientific, USA), employing the PerfectStart Green qPCR SuperMix (TransGen, China) as per manufacturer's instructions. Data normalization was achieved by utilizing an endogenous control (actin) and intracellular mRNA or circRNA quantities. Table S1 provides a comprehensive list of all primers employed for amplification in RT-qPCR assays.

### Encapsulation of circRNA by LNP

LNP@circRNA were prepared by combining an aqueous phase containing circRNA with an ethanol phase containing the lipids and cholesterol components *via* microfluidic mixing devices. The devices utilized chaotic mixing features to induce fluid folding in a state of laminar flow to reproducibly form homogeneous LNP. The aqueous phase was composed of 100 mM citrate buffer (pH = 5.0) and circRNAs. The ethanol phase contained the ionizable lipid, 1,2-distearoyl-sn-glycerol-3-phosphocholine, cholesterol, and lipid-polyethylene glycol at the molar ratio of 50:10:38:2. The aqueous and ethanol phases were then mixed in the microfluidic device at a 1:3 (*v*/*v*) ratio. After preparation, the LNP@circRNA were dialyzed against PBS for 12 hours (MWCO = 3.5 kDa). To measure size, the LNP@circRNA were 10 times diluted with PBS and analyzed using DLS performed on a Zetasizer Nano (Malvern Instruments, UK). The diameter and polydispersity index of the LNP were measured in triplicate. To measure the morphology, LNP@circRNA were dialyzed against deionized water for 2 hours (MWCO = 3.5 kDa) twice. Then the dialyzed LNP@circRNA was diluted 100 times using deionized water and 10 μL of the sample was dropped onto a carbon-coated copper grid. Samples were dried under a vacuum to remove water. Tests were carried out on a Hitachi HT-7700 (Hitachi, Japan).

### Mouse immunization and tumor model

The female BALB/c and C57BL/6J mice (aged 6 to 8 weeks) were immunized intramuscularly twice with LNP@circRNA vaccine containing 10 µg of circRNA. For the tumor model, subcutaneous injection of 5x10^5^ TC-1cells was performed on the right flank of C57BL/6J mice. Tumor volumes were measured twice a week using a caliper by assessing the length (a), width (b) and height (h), and calculated according to the formula: tumor volume = abh/2.

### Flow cytometry analysis

Spleens from mice were processed to obtain single cell suspensions, and 2 x10^6^ cells per sample were utilized for flow cytometry analysis. To prevent non-specific binding, the cells were incubated with anti-FcgIII/II receptor (clone 2.4G2) for a duration of 20 minutes. Fixable Viability Dye eFluor™ 450 (eBioscience, USA) was employed to exclude dead cells from the analysis. APC anti-mouse CD8α (BioLegend, USA) was used for staining CD8^+^ T cells, while PE anti-mouse IFN-γ (BioLegend, USA) was intracellularly stained using True-Nuclear^TM^ transcription factor buffer set (BioLegend, USA), following the manufacturer's instructions. Data acquisition was performed on a CytoFLEX flow cytometer (Beckman Coulter, USA), and subsequent analysis was conducted using CytExpert software (BeckmanCoulter, USA).

### Enzyme-linked immunosorbent assay (ELISA)

ELISA plates (Corning, USA) were coated overnight with 2 µg/mL of monomeric RBD protein from SARS-CoV-2 and its variant in a carbonate-bicarbonate buffer (0.05 M, pH 9.6), followed by blocking with 5% skim milk in PBS. Serum samples were diluted and added to each well. Plates were incubated with goat anti-mouse IgG-HRP antibody and developed with 3,3',5,5'-tetramethylbenzidine (TMB) substrate. Reactions were stopped with 2 M hydrochloric acid, and the absorbance was measured at 450 nm. The endpoint titer was defined as the highest reciprocal dilution of serum to give an absorbance greater than 2.5-fold of the background values. Antibody titer below the limit of detection was determined as half the limit of detection.

### Statistical analysis

Survival curves were analyzed using Kaplan-Meier analysis and its *P* value was determined by log-rank test. Unpaired two-tailed *t*-tests were employed for all other data analyses. A value of *P* < 0.05 was considered statistically significant.

## Supplementary Material

Supplementary figures and table.

## Abbreviations

CircRNA: circular RNA
mRNA: messenger RNA
PIE: permuted intron-exon
HPLC: high-performance liquid chromatography
HPV: human papillomavirus
dRBD: dimerized receptor binding domains
LNP: lipid nanoparticle
CPIE: chimeric permuted intron-exon system
Td: thymidylate
ssRNA: single-stranded RNA
BrCN: cyanogen bromide
EDC: 1-ethyl-3-(3'-dimethylaminopropyl) carbodiimide
dsRNA: double-stranded RNA
RT-PCR: reverse transcription-PCR
D2GFP: destabilized green fluorescent protein
PEI: polyethylenimine
RT-qPCR: quantitative reverse transcription PCR
SP: signal peptide
ELISA: enzyme-linked immunosorbent assay
IRES: internal ribosome entry site
CVB3: Coxsackievirus B3
EGFP: enhanced green fluorescent protein
DLS: dynamic light scattering
TEM: transmission electron microscopy
