# Supplementary material for: Developing an enhanced chimeric permuted intron-exon system for circular RNA therapeutics
Source: Theranostics. 2024 Sep 9;14(15):5869–82. doi: 10.7150/thno.98214 (PMC11426236; doi:10.7150/thno.98214)
Supplement: Supplementary file 1 — Supplementary figures and table. [file thnov14p5869s1.pdf]

Developing an enhanced chimeric permuted intron-exon system for circular RNA therapeutics

Lei Wang[1,2,3,†], Chunbo Dong[1,2,4,†], Weibing Zhang[4], Xu Ma[4], Wei Rou[2], Kai Yang[5], Tong Cui[2], Shaolong Qi[5], Lijun Yang[1,3], Jun Xie[1,3], Guocan Yu[5,*], Lianqing Wang[6,*], Xiaoyuan Chen[7,8,9,10,11,*], Zhida Liu[1,2,4,*]

[1]MOE Key Laboratory of Coal Environmental Pathogenicity and Prevention, Shanxi Medical University, Taiyuan 030001, China
[2]Shanxi Academy of Advanced Research and Innovation, Taiyuan 030032, China
[3]Department of Biochemistry and Molecular Biology, Shanxi Key Laboratory of Birth Defect and Cell Regeneration, Shanxi Medical University, Taiyuan 030001, China
[4]College of Veterinary Medicine, Shanxi Agricultural University, Jinzhong 030801, China
[5]Key Laboratory of Bioorganic Phosphorus Chemistry & Chemical Biology, Department of Chemistry, Tsinghua University, Beijing 100084, China
[6]Center of Translational Medicine, Zibo Central Hospital, Zibo 255036, China
[7]Departments of Diagnostic Radiology, Surgery, Chemical and Biomolecular Engineering, and Biomedical Engineering, Yong Loo Lin School of Medicine and College of Design and Engineering, National University of Singapore, Singapore 119074, Singapore
[8]Clinical Imaging Research Centre, Centre for Translational Medicine, Yong Loo Lin School of Medicine, National University of Singapore, Singapore 117599, Singapore
[9]Nanomedicine Translational Research Program, Yong Loo Lin School of Medicine, National University of Singapore, Singapore 117597, Singapore
[10]Theranostics Center of Excellence (TCE), Yong Loo Lin School of Medicine, National University of Singapore, Singapore 138667, Singapore
[11]Institute of Molecular and Cell Biology, Agency for Science, Technology, and Research (A*STAR), Singapore 138673, Singapore

[†]These authors contributed equally: Lei Wang, Chunbo Dong

*Correspondence:
Zhida Liu (Zhida_Liu@saari.org.cn)
Xiaoyuan Chen (chen.shawn@nus.edu.sg)
Lianqing Wang (lianqing.wang@hotmail.com)
Guocan Yu (guocanyu@mail.tsinghua.edu.cn)

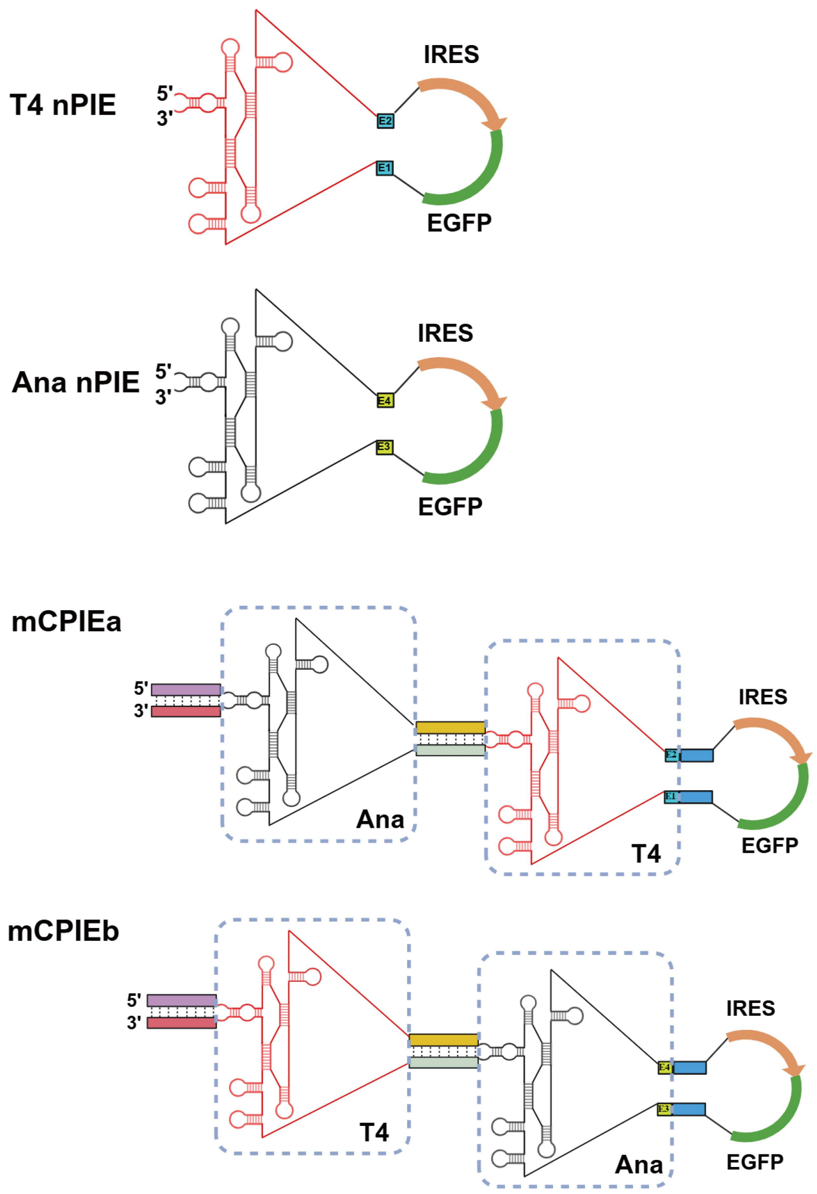

Figure S1. A schematic diagram depicts the components of the T4-nPIE, Ana-nPIE, mCPIEa, and mCPIEb systems.

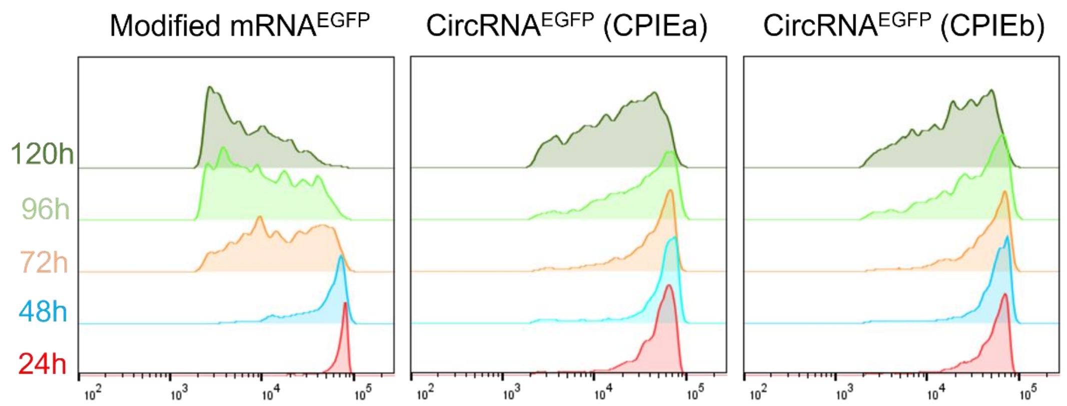

Figure S2. Assessment of EGFP expression in 293F cells transfected with circRNA^{EGFP} (CPIEa), circRNA^{EGFP} (CPIEb) and modified mRNA^{EGFP} over 5 days by flow cytometry.

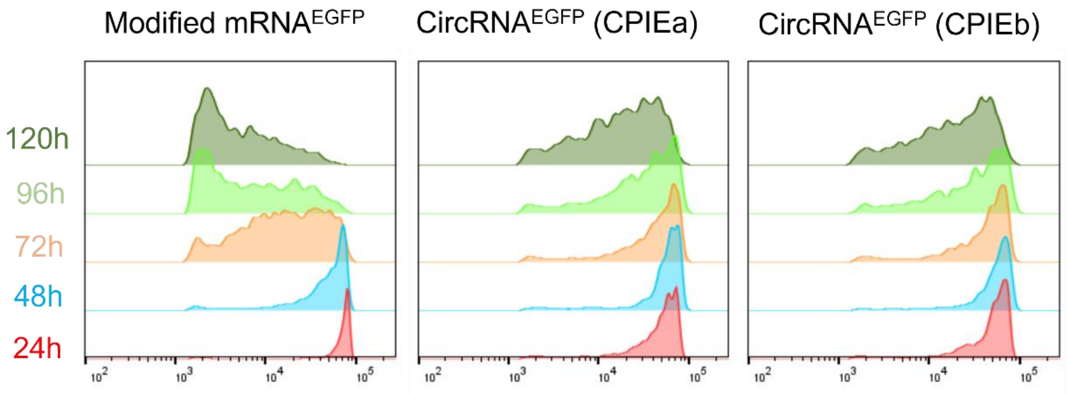

Figure S3. Assessment of EGFP expression in 293T cells transfected with circRNA^{EGFP} (CPIEa), circRNA^{EGFP} (CPIEb) and modified mRNA^{EGFP} over 5 days by flow cytometry.

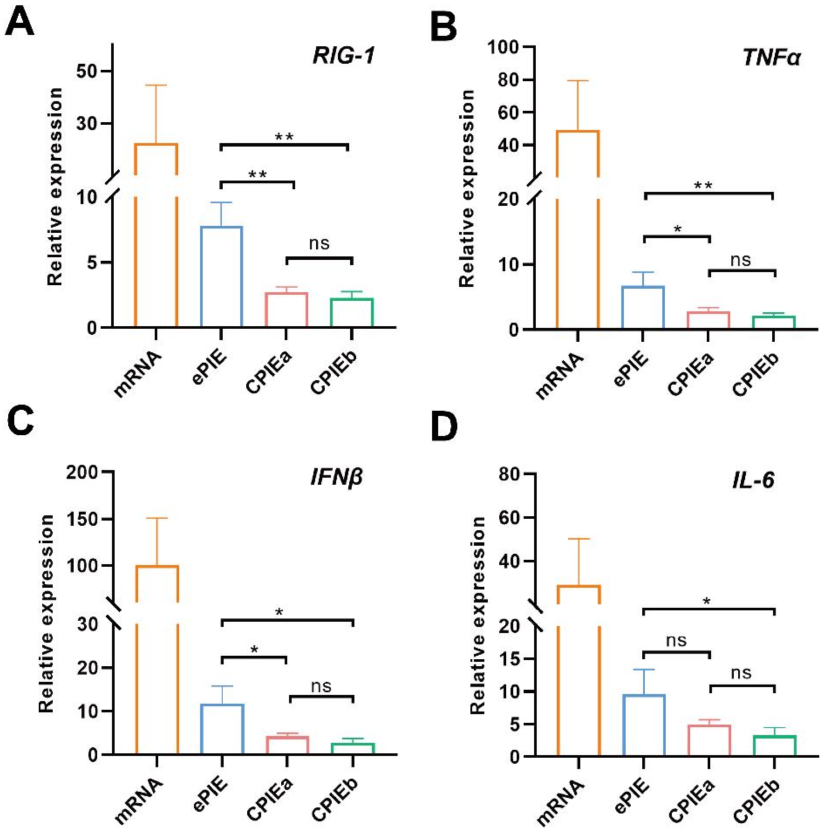

Figure S4. RT-qPCR analysis was performed to evaluate the relative expression of specific genes, *RIG-1* (a), *TNFα* (b), *IFNβ* (c) and *IL-6* (d) in A549 cells transfected with ePIE- and CPIE-generated $circRNA^{EGFP}$ at 12 h post-transfection. Notably, the unmodified linear $mRNA^{EGFP}$ served as control. The mRNA expression levels were normalized not only using actin as a reference gene, but also by considering intracellular mRNA or circRNA quantities to account for variations in transfection efficiencies and RNA stability.

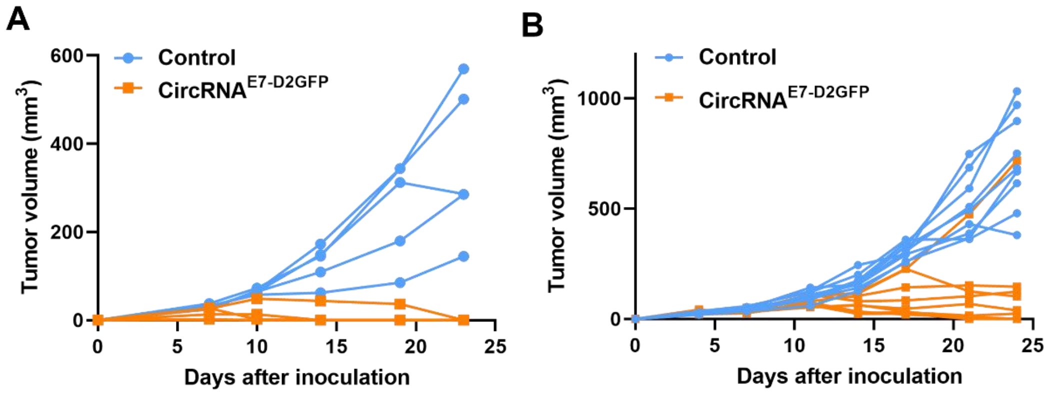

Figure S5. Individual tumor growth curves of the mice from control and vaccinated groups in Figure 6B and Figure 6D.

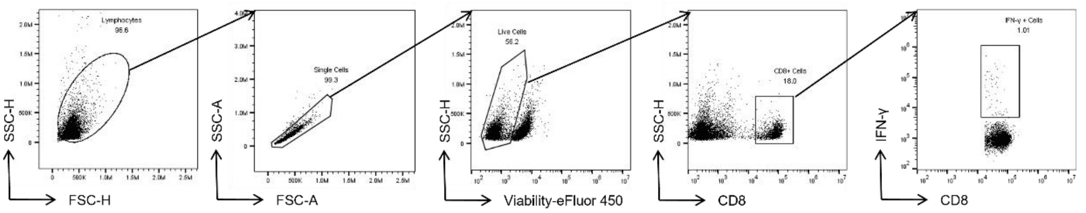

Figure S6. Flow cytometric gating strategies for the identification of IFN-γ^{+}CD8^{+} T cells subset.

Table S1

IVT templetes	
CircRNA^{EGFP} templete (T4 nPIE)	GGTTCTACATAAATGCCTAACGACTATCCCTTTGGGGAGTAGGGTCAAGTGACTCGA<br>AACGATAGACAACTTGCTTTAACAAGTTGGAGATATAGTCTGCTCTGCATGGTGACA<br>TGCAGCTGGATATAATTCCGGGGTAAGATTAACGACCTTATCTGAACATAATGCTACC<br>GTTTAATATTGCGTCATTTAAAACAGCCTGTGGGTTGATCCCACCCACAGGCCCATT<br>GGGCGCTAGCACTCTGGTATCACGGTACCTTTGTGCGCCTGTTTTATACCCCCTCCC<br>CCAACTGTAACTTAGAAGTAACACACACCGATCAACAGTCAGCGTGGCACACCAG<br>CCACGTTTTGATCAAGCACTTCTGTTACCCCGGACTGAGTATCAATAGACTGCTCAC<br>GCGGTTGAAGGAGAAAGCGTTCGTTATCCGGCCAACTACTTCGAAAAACCTAGTAA<br>CACCGTGGAAGTTGCAGAGTGTTTCGCTCAGCACTACCCCAGTGTAGATCAGGTCG<br>ATGAGTCACCGCATTCCCCACGGGCGACCGTGGCGGTGGCTGCGTTGGCGGCCTGC<br>CCATGGGGAAACCCATGGGACGCTCTAATACAGACATGGTGCGAAGAGTCTATTGA<br>GCTAGTTGGTAGTCCTCCGGCCCCTGAATGCGGCTAATCCTAACTGCGGAGCACAC<br>ACCCTCAAGCCAGAGGGCAGTGTGTCGTAACGGGCAACTCTGCAGCGGAACCGAC<br>TACTTTGGGTGTCCGTGTTTCATTTTATTCCTATACTGGCTGCTTATGGTGACAATTG<br>AGAGATCGTTACCATATAGCTATTGGATTGGCCATCCGGTGACTAATAGAGCTATTAT<br>ATATCCCTTTGTTGGGTTTATACCACTTAGCTTGAAAGAGGTTAAAACATTACAATTC<br>ATTGTTAAGTTGAATACAGCAAAGCCACCATGGTGAGCAAGGGCGAGGAGCTGTTC<br>ACCGGGGTGGTGCCCATCCTGGTCGAGCTGGACGGCGACGTAAACGGCCACAAGT<br>TCAGCGTGTCCGGCGAGGGCGAGGGCGATGCCACCTACGGCAAGCTGACCCTGAA<br>GTTCATCTGCACCACCGGCAAGCTGCCCGTGCCCTGGCCCACCCTCGTGACCACCC<br>TGACCTACGGCGTGCAGTGCTTCAGCCGCTACCCCGACCACATGAAGCAGCACGAC<br>TTCTTCAAGTCCGCCATGCCCGAAGGCTACGTCCAGGAGCGCACCATCTTCTTCAA<br>GGACGACGGCAACTACAAGACCCGCGCCGAGGTGAAGTTCGAGGGCGACACCCT<br>GGTGAACCGCATCGAGCTGAAGGGCATCGACTTCAAGGAGGACGGCAACATCCTG<br>GGGCACAAGCTGGAGTACAACTACAACAGCCACAACGTCTATATCATGGCCGACAA<br>GCAGAAGAACGGCATCAAGGTGAACTTCAAGATCCGCCACAACATCGAGGACGGC<br>AGCGTGCAGCTCGCCGACCACTACCAGCAGAACACCCCCATCGGCGACGGCCCCG<br>TGCTGCTGCCCGACAACCACTACCTGAGCACCCAGTCCGCCCTGAGCAAAGACCC<br>CAACGAGAAGCGCGATCACATGGTCCTGCTGGAGTTCGTGACCGCCGCCGGGATC<br>ACTCTCGGCATGGACGAGCTGTACAAGTAAAGATGTTTTCTTGGGTTAATTGAGGC<br>CTGAGTATAAGGTGACTTATACTTGTAATCTATCTAAACGGGGAACCTCTCTAGTAG<br>ACAATCCCGTGCTAAATTGTAGGACT
CircRNA^{EGFP} templete (Ana nPIE)	TGACTTACAACTAATCGGAAGGTGCAGAGACTCGACGGGAGCTACCCTAACGTCAA<br>GACGAGGGTAAAGAGAGAGTCCAATTCTCAAAGCCAATAGGCAGTAGCGAAAGCT<br>GCAAGAGAATGAAAATCCGTTTAAAACAGCCTGTGGGTTGATCCCACCCACAGGCC<br>CATTGGGCGCTAGCACTCTGGTATCACGGTACCTTTGTGCGCCTGTTTTATACCCCCT<br>CCCCCAACTGTAACTTAGAAGTAACACACACCGATCAACAGTCAGCGTGGCACACC<br>AGCCACGTTTTGATCAAGCACTTCTGTTACCCCGGACTGAGTATCAATAGACTGCTC<br>ACGCGGTTGAAGGAGAAAGCGTTCGTTATCCGGCCAACTACTTCGAAAAACCTAGT<br>AACACCGTGGAAGTTGCAGAGTGTTTCGCTCAGCACTACCCCAGTGTAGATCAGGT<br>CGATGAGTCACCGCATTCCCCACGGGCGACCGTGGCGGTGGCTGCGTTGGCGGCCT<br>GCCCATGGGGAAACCCATGGGACGCTCTAATACAGACATGGTGCGAAGAGTCTATT

	GAGCTAGTTGGTAGTCCTCCGGCCCCTGAATGCGGCTAATCCTAACTGCGGAGCAC<br>ACACCCTCAAGCCAGAGGGCAGTGTGTCGTAACGGGCAACTCTGCAGCGGAACCG<br>ACTACTTTGGGTGTCCGTGTTTCATTTTATTCCTATACTGGCTGCTTATGGTGACAAT<br>TGAGAGATCGTTACCATATAGCTATTGGATTGGCCATCCGGTGACTAATAGAGCTATT<br>ATATATCCCTTTGTTGGGTTTATACCACTTAGCTTGAAAGAGGTTAAAACATTACAAT<br>TCATTGTTAAGTTGAATACAGCAAAGCCACCATGGTGAGCAAGGGCGAGGAGCTGT<br>TCACCGGGGTGGTGCCCATCCTGGTCGAGCTGGACGGCGACGTAAACGGCCACAA<br>GTTCAGCGTGTCCGGCGAGGGCGAGGGCGATGCCACCTACGGCAAGCTGACCCTG<br>AAGTTCATCTGCACCACCGGCAAGCTGCCCGTGCCCTGGCCCACCCTCGTGACCAC<br>CCTGACCTACGGCGTGCAGTGCTTCAGCCGCTACCCCGACCACATGAAGCAGCACG<br>ACTTCTTCAAGTCCGCCATGCCCGAAGGCTACGTCCAGGAGCGCACCATCTTCTTC<br>AAGGACGACGGCAACTACAAGACCCGCGCCGAGGTGAAGTTCGAGGGCGACACC<br>CTGGTGAACCGCATCGAGCTGAAGGGCATCGACTTCAAGGAGGACGGCAACATCC<br>TGGGGCACAAGCTGGAGTACAACTACAACAGCCACAACGTCTATATCATGGCCGAC<br>AAGCAGAAGAACGGCATCAAGGTGAACTTCAAGATCCGCCACAACATCGAGGACG<br>GCAGCGTGCAGCTCGCCGACCACTACCAGCAGAACACCCCCATCGGCGACGGCCC<br>CGTGCTGCTGCCCGACAACCACTACCTGAGCACCCAGTCCGCCCTGAGCAAAGAC<br>CCCAACGAGAAGCGCGATCACATGGTCCTGCTGGAGTTCGTGACCGCCGCCGGGAT<br>CACTCTCGGCATGGACGAGCTGTACAAGTAAACGGACTTAAATAATTGAGCCTTAA<br>AGAAGAAATTCTTTAAGTGGATGCTCTCAAACTCAGGGAAACCTAAATCTAGTTATA<br>GACAAGGCAATCCTGAGCCAAGCCGAAGTAGTAATTAGTAAGTTA
$CircRNA^{EGFP}$ templete (mCPIEa)	TTATAATGTGGATGTATATATGACTTACAACTAATCGGAAGGTGCAGAGACTCGACG<br>GGAGCTACCCTAACGTCAAGACGAGGGTAAAGAGAGAGTCCAATTCTCAAAGCCA<br>ATAGGCAGTAGCGAAAGCTGCAAGAGAATGTGGATTGGCAGCCAGACTGCGGTTCT<br>ACATAAATGCCTAACGACTATCCCTTTGGGGAGTAGGGTCAAGTGACTCGAAACGA<br>TAGACAACTTGCTTTAACAAGTTGGAGATATAGTCTGCTCTGCATGGTGACATGCAG<br>CTGGATATAATTCCGGGGTAAGATTAACGACCTTATCTGAACATAATGCTACCGTTTA<br>ATATTGCGTCATAAAAAACAAAAAACAAAAAACAAAACAAAATTAAAACAGCCTG<br>TGGGTTGATCCCACCCACAGGCCCATTGGGCGCTAGCACTCTGGTATCACGGTACCT<br>TTGTGCGCCTGTTTTATACCCCCTCCCCCAACTGTAACTTAGAAGTAACACACACCG<br>ATCAACAGTCAGCGTGGCACACCAGCCACGTTTTGATCAAGCACTTCTGTTACCCC<br>GGACTGAGTATCAATAGACTGCTCACGCGGTTGAAGGAGAAAGCGTTCGTTATCCG<br>GCCAACTACTTCGAAAAACCTAGTAACACCGTGGAAGTTGCAGAGTGTTTCGCTCA<br>GCACTACCCCAGTGTAGATCAGGTCGATGAGTCACCGCATTCCCCACGGGCGACCG<br>TGGCGGTGGCTGCGTTGGCGGCCTGCCCATGGGGAAACCCATGGGACGCTCTAATA<br>CAGACATGGTGCGAAGAGTCTATTGAGCTAGTTGGTAGTCCTCCGGCCCCTGAATG<br>CGGCTAATCCTAACTGCGGAGCACACACCCTCAAGCCAGAGGGCAGTGTGTCGTA<br>ACGGGCAACTCTGCAGCGGAACCGACTACTTTGGGTGTCCGTGTTTCATTTTATTCC<br>TATACTGGCTGCTTATGGTGACAATTGAGAGATCGTTACCATATAGCTATTGGATTGG<br>CCATCCGGTGACTAATAGAGCTATTATATATCCCTTTGTTGGGTTTATACCACTTAGCT<br>TGAAAGAGGTTAAAACATTACAATTCATTGTTAAGTTGAATACAGCAAAGCCACCAT<br>GGTGAGCAAGGGCGAGGAGCTGTTCACCGGGGTGGTGCCCATCCTGGTCGAGCTG<br>GACGGCGACGTAAACGGCCACAAGTTCAGCGTGTCCGGCGAGGGCGAGGGCGATG<br>CCACCTACGGCAAGCTGACCCTGAAGTTCATCTGCACCACCGGCAAGCTGCCCGTG

	CCCTGGCCCACCCTCGTGACCACCCTGACCTACGGCGTGCAGTGCTTCAGCCGCTA<br>CCCCGACCACATGAAGCAGCACGACTTCTTCAAGTCCGCCATGCCCGAAGGCTACG<br>TCCAGGAGCGCACCATCTTCTTCAAGGACGACGGCAACTACAAGACCCGCGCCGA<br>GGTGAAGTTCGAGGGCGACACCCTGGTGAACCGCATCGAGCTGAAGGGCATCGAC<br>TTCAAGGAGGACGGCAACATCCTGGGGCACAAGCTGGAGTACAACTACAACAGCC<br>ACAACGTCTATATCATGGCCGACAAGCAGAAGAACGGCATCAAGGTGAACTTCAAG<br>ATCCGCCACAACATCGAGGACGGCAGCGTGCAGCTCGCCGACCACTACCAGCAGA<br>ACACCCCCATCGGCGACGGCCCCGTGCTGCTGCCCGACAACCACTACCTGAGCACC<br>CAGTCCGCCCTGAGCAAAGACCCCAACGAGAAGCGCGATCACATGGTCCTGCTGG<br>AGTTCGTGACCGCCGCCGGGATCACTCTCGGCATGGACGAGCTGTACAAGTAAAAA<br>AAAAAAAAAAAAAAAAAAGATGTTTTCTTGGGTTAATTGAGGCCTGAGTATAAGGT<br>GACTTATACTTGTAATCTATCTAAACGGGGAACCTCTCTAGTAGACAATCCCGTGCT<br>AAATTGTAGGACTGCAGTCTGGCTGCCAATCCAAAATAATTGAGCCTTAAAGAAGA<br>AATTCTTTAAGTGGATGCTCTCAAACTCAGGGAAACCTAAATCTAGTTATAGACAAG<br>GCAATCCTGAGCCAAGCCGAAGTAGTAATTAGTAAGTTATATATGCGTTCATATTATA<br>G
CircRNA^{EGFP} templete (mCPIEb)	TTATAATGTGGATGTATATAGGTTCTACATAAATGCCTAACGACTATCCCTTTGGGGA<br>GTAGGGTCAAGTGACTCGAAACGATAGACAACTTGCTTTAACAAGTTGGAGATATA<br>GTCTGCTCTGCATGGTGACATGCAGCTGGATATAATTCCGGGGTAAGATTAACGACC<br>TTATCTGAACATAATGTGGATTGGCAGCCAGACTGCTGACTTACAACTAATCGGAAG<br>GTGCAGAGACTCGACGGGAGCTACCCTAACGTCAAGACGAGGGTAAAGAGAGAGT<br>CCAATTCTCAAAGCCAATAGGCAGTAGCGAAAGCTGCAAGAGAATGAAAATCCGTA<br>AAAAACAAAAAACAAAAAACAAAACAAAATTAAAACAGCCTGTGGGTTGATCCCA<br>CCCACAGGCCCATTGGGCGCTAGCACTCTGGTATCACGGTACCTTTGTGCGCCTGTT<br>TTATACCCCCTCCCCCAACTGTAACTTAGAAGTAACACACACCGATCAACAGTCAG<br>CGTGGCACACCAGCCACGTTTTGATCAAGCACTTCTGTTACCCCGGACTGAGTATC<br>AATAGACTGCTCACGCGGTTGAAGGAGAAAGCGTTCGTTATCCGGCCAACTACTTC<br>GAAAAACCTAGTAACACCGTGGAAGTTGCAGAGTGTTTCGCTCAGCACTACCCCA<br>GTGTAGATCAGGTCGATGAGTCACCGCATTCCCCACGGGCGACCGTGGCGGTGGCT<br>GCGTTGGCGGCCTGCCCATGGGGAAACCCATGGGACGCTCTAATACAGACATGGTG<br>CGAAGAGTCTATTGAGCTAGTTGGTAGTCCTCCGGCCCCTGAATGCGGCTAATCCTA<br>ACTGCGGAGCACACACCCTCAAGCCAGAGGGCAGTGTGTCGTAACGGGCAACTCT<br>GCAGCGGAACCGACTACTTTGGGTGTCCGTGTTTCATTTTATTCCTATACTGGCTGCT<br>TATGGTGACAATTGAGAGATCGTTACCATATAGCTATTGGATTGGCCATCCGGTGACT<br>AATAGAGCTATTATATATCCCTTTGTTGGGTTTATACCACTTAGCTTGAAAGAGGTTA<br>AAACATTACAATTCATTGTTAAGTTGAATACAGCAAAGCCACCATGGTGAGCAAGG<br>GCGAGGAGCTGTTCACCGGGGTGGTGCCCATCCTGGTCGAGCTGGACGGCGACGT<br>AAACGGCCACAAGTTCAGCGTGTCCGGCGAGGGCGAGGGCGATGCCACCTACGGC<br>AAGCTGACCCTGAAGTTCATCTGCACCACCGGCAAGCTGCCCGTGCCCTGGCCCAC<br>CCTCGTGACCACCCTGACCTACGGCGTGCAGTGCTTCAGCCGCTACCCCGACCACA<br>TGAAGCAGCACGACTTCTTCAAGTCCGCCATGCCCGAAGGCTACGTCCAGGAGCG<br>CACCATCTTCTTCAAGGACGACGGCAACTACAAGACCCGCGCCGAGGTGAAGTTC<br>GAGGGCGACACCCTGGTGAACCGCATCGAGCTGAAGGGCATCGACTTCAAGGAGG<br>ACGGCAACATCCTGGGGCACAAGCTGGAGTACAACTACAACAGCCACAACGTCTAT

	ATCATGGCCGACAAGCAGAAGAACGGCATCAAGGTGAACTTCAAGATCCGCCACA<br>ACATCGAGGACGGCAGCGTGCAGCTCGCCGACCACTACCAGCAGAACACCCCCAT<br>CGGCGACGGCCCCGTGCTGCTGCCCGACAACCACTACCTGAGCACCCAGTCCGCC<br>CTGAGCAAAGACCCCAACGAGAAGCGCGATCACATGGTCCTGCTGGAGTTCGTGA<br>CCGCCGCCGGGATCACTCTCGGCATGGACGAGCTGTACAAGTAAAAAAAAAAAAA<br>AAAAAAAAAACGGACTTAAATAATTGAGCCTTAAAGAAGAAATTCTTTAAGTGGAT<br>GCTCTCAAACTCAGGGAAACCTAAATCTAGTTATAGACAAGGCAATCCTGAGCCAA<br>GCCGAAGTAGTAATTAGTAAGTTAGCAGTCTGGCTGCCAATCCATAATTGAGGCCTG<br>AGTATAAGGTGACTTATACTTGTAATCTATCTAAACGGGGAACCTCTCTAGTAGACA<br>ATCCCGTGCTAAATTGTAGGACTTATATGCGTTCATATTATAG
CircRNA^{EGFP} templete (CPIEa)	TTATAATGTGGATGTATATATGACTTACAACTAATCGGAAGGTGCAGAGACTCGACG<br>GGAGCTACCCTAACGTCAAGACGAGGGTAAAGAGAGAGTCCAATTCTCAAAGCCA<br>ATAGGCAGTAGCGAAAGCTGCAAGAGAATGAAAATCCGTTGGATTGGCAGCCAGA<br>CTGCGGTTCTACATAAATGCCTAACGACTATCCCTTTGGGGAGTAGGGTCAAGTGAC<br>TCGAAACGATAGACAACTTGCTTTAACAAGTTGGAGATATAGTCTGCTCTGCATGGT<br>GACATGCAGCTGGATATAATTCCGGGGTAAGATTAACGACCTTATCTGAACATAATG<br>CTACCGTTTAATATTGCGTCATAAAAAACAAAAAACAAAAAACAAAACAAAATTAA<br>AACAGCCTGTGGGTTGATCCCACCCACAGGCCCATTGGGCGCTAGCACTCTGGTAT<br>CACGGTACCTTTGTGCGCCTGTTTTATACCCCCTCCCCCAACTGTAACTTAGAAGTA<br>ACACACACCGATCAACAGTCAGCGTGGCACACCAGCCACGTTTTGATCAAGCACTT<br>CTGTTACCCCGGACTGAGTATCAATAGACTGCTCACGCGGTTGAAGGAGAAAGCGT<br>TCGTTATCCGGCCAACTACTTCGAAAAACCTAGTAACACCGTGGAAGTTGCAGAGT<br>GTTTCGCTCAGCACTACCCCAGTGTAGATCAGGTCGATGAGTCACCGCATTCCCCAC<br>GGGCGACCGTGGCGGTGGCTGCGTTGGCGGCCTGCCCATGGGGAAACCCATGGGA<br>CGCTCTAATACAGACATGGTGCGAAGAGTCTATTGAGCTAGTTGGTAGTCCTCCGGC<br>CCCTGAATGCGGCTAATCCTAACTGCGGAGCACACACCCTCAAGCCAGAGGGCAGT<br>GTGTCGTAACGGGCAACTCTGCAGCGGAACCGACTACTTTGGGTGTCCGTGTTTCA<br>TTTTATTCCTATACTGGCTGCTTATGGTGACAATTGAGAGATCGTTACCATATAGCTAT<br>TGGATTGGCCATCCGGTGACTAATAGAGCTATTATATATCCCTTTGTTGGGTTTATACC<br>ACTTAGCTTGAAAGAGGTTAAAACATTACAATTCATTGTTAAGTTGAATACAGCAAA<br>GCCACCATGGTGAGCAAGGGCGAGGAGCTGTTCACCGGGGTGGTGCCCATCCTGG<br>TCGAGCTGGACGGCGACGTAAACGGCCACAAGTTCAGCGTGTCCGGCGAGGGCGA<br>GGGCGATGCCACCTACGGCAAGCTGACCCTGAAGTTCATCTGCACCACCGGCAAG<br>CTGCCCGTGCCCTGGCCCACCCTCGTGACCACCCTGACCTACGGCGTGCAGTGCTT<br>CAGCCGCTACCCCGACCACATGAAGCAGCACGACTTCTTCAAGTCCGCCATGCCCG<br>AAGGCTACGTCCAGGAGCGCACCATCTTCTTCAAGGACGACGGCAACTACAAGAC<br>CCGCGCCGAGGTGAAGTTCGAGGGCGACACCCTGGTGAACCGCATCGAGCTGAAG<br>GGCATCGACTTCAAGGAGGACGGCAACATCCTGGGGCACAAGCTGGAGTACAACT<br>ACAACAGCCACAACGTCTATATCATGGCCGACAAGCAGAAGAACGGCATCAAGGT<br>GAACTTCAAGATCCGCCACAACATCGAGGACGGCAGCGTGCAGCTCGCCGACCAC<br>TACCAGCAGAACACCCCCATCGGCGACGGCCCCGTGCTGCTGCCCGACAACCACTA<br>CCTGAGCACCCAGTCCGCCCTGAGCAAAGACCCCAACGAGAAGCGCGATCACATG<br>GTCCTGCTGGAGTTCGTGACCGCCGCCGGGATCACTCTCGGCATGGACGAGCTGTA<br>CAAGTAAAAAAAAAAAAAAAAAAAAAAAAAGATGTTTTCTTGGGTTAATTGAGGCCT

	GAGTATAAGGTGACTTATACTTGTAATCTATCTAAACGGGGAACCTCTCTAGTAGAC<br>AATCCCGTGCTAAATTGTAGGACTGCAGTCTGGCTGCCAATCCAACGGACTTAAATA<br>ATTGAGCCTTAAAGAAGAAATTCTTTAAGTGGATGCTCTCAAACTCAGGGAAACCT<br>AAATCTAGTTATAGACAAGGCAATCCTGAGCCAAGCCGAAGTAGTAATTAGTAAGTT<br>ATATATGCGTTCATATTATAG
CircRNA^{EGFP} templete (CPIEb)	TTATAATGTGGATGTATATAGGTTCTACATAAATGCCTAACGACTATCCCTTTGGGGA<br>GTAGGGTCAAGTGACTCGAAACGATAGACAACTTGCTTTAACAAGTTGGAGATATA<br>GTCTGCTCTGCATGGTGACATGCAGCTGGATATAATTCCGGGGTAAGATTAACGACC<br>TTATCTGAACATAATGCTACCGTTTAATATTGCGTCATTGGATTGGCAGCCAGACTGC<br>TGACTTACAACTAATCGGAAGGTGCAGAGACTCGACGGGAGCTACCCTAACGTCAA<br>GACGAGGGTAAAGAGAGAGTCCAATTCTCAAAGCCAATAGGCAGTAGCGAAAGCT<br>GCAAGAGAATGAAAATCCGTAAAAAACAAAAAACAAAAAACAAAACAAAATTAA<br>AACAGCCTGTGGGTTGATCCCACCCACAGGCCCATTGGGCGCTAGCACTCTGGTAT<br>CACGGTACCTTTGTGCGCCTGTTTTATACCCCCTCCCCCAACTGTAACTTAGAAGTA<br>ACACACACCGATCAACAGTCAGCGTGGCACACCAGCCACGTTTTGATCAAGCACTT<br>CTGTTACCCCGGACTGAGTATCAATAGACTGCTCACGCGGTTGAAGGAGAAAGCGT<br>TCGTTATCCGGCCAACTACTTCGAAAAACCTAGTAACACCGTGGAAGTTGCAGAGT<br>GTTTCGCTCAGCACTACCCCAGTGTAGATCAGGTCGATGAGTCACCGCATTCCCCAC<br>GGGCGACCGTGGCGGTGGCTGCGTTGGCGGCCTGCCCATGGGGAAACCCATGGGA<br>CGCTCTAATACAGACATGGTGCGAAGAGTCTATTGAGCTAGTTGGTAGTCCTCCGGC<br>CCCTGAATGCGGCTAATCCTAACTGCGGAGCACACACCCTCAAGCCAGAGGGCAGT<br>GTGTCGTAACGGGCAACTCTGCAGCGGAACCGACTACTTTGGGTGTCCGTGTTTCA<br>TTTTATTCCTATACTGGCTGCTTATGGTGACAATTGAGAGATCGTTACCATATAGCTAT<br>TGGATTGGCCATCCGGTGACTAATAGAGCTATTATATATCCCTTTGTTGGGTTTATACC<br>ACTTAGCTTGAAAGAGGTTAAAACATTACAATTCATTGTTAAGTTGAATACAGCAAA<br>GCCACCATGGTGAGCAAGGGCGAGGAGCTGTTCACCGGGGTGGTGCCCATCCTGG<br>TCGAGCTGGACGGCGACGTAAACGGCCACAAGTTCAGCGTGTCCGGCGAGGGCGA<br>GGGCGATGCCACCTACGGCAAGCTGACCCTGAAGTTCATCTGCACCACCGGCAAGC<br>TGCCCGTGCCCTGGCCCACCCTCGTGACCACCCTGACCTACGGCGTGCAGTGCTTC<br>AGCCGCTACCCCGACCACATGAAGCAGCACGACTTCTTCAAGTCCGCCATGCCCGA<br>AGGCTACGTCCAGGAGCGCACCATCTTCTTCAAGGACGACGGCAACTACAAGACCC<br>GCGCCGAGGTGAAGTTCGAGGGCGACACCCTGGTGAACCGCATCGAGCTGAAGGG<br>CATCGACTTCAAGGAGGACGGCAACATCCTGGGGCACAAGCTGGAGTACAACTAC<br>AACAGCCACAACGTCTATATCATGGCCGACAAGCAGAAGAACGGCATCAAGGTGA<br>ACTTCAAGATCCGCCACAACATCGAGGACGGCAGCGTGCAGCTCGCCGACCACTAC<br>CAGCAGAACACCCCCATCGGCGACGGCCCCGTGCTGCTGCCCGACAACCACTACCT<br>GAGCACCCAGTCCGCCCTGAGCAAAGACCCCAACGAGAAGCGCGATCACATGGTC<br>CTGCTGGAGTTCGTGACCGCCGCCGGGATCACTCTCGGCATGGACGAGCTGTACAA<br>GTAAAAAAAAAAAAAAAAAAAAAAACGGACTTAAATAATTGAGCCTTAAAGAAGA<br>AATTCTTTAAGTGGATGCTCTCAAACTCAGGGAAACCTAAATCTAGTTATAGACAAG<br>GCAATCCTGAGCCAAGCCGAAGTAGTAATTAGTAAGTTAGCAGTCTGGCTGCCAAT<br>CCAAGATGTTTTCTTGGGTTAATTGAGGCCTGAGTATAAGGTGACTTATACTTGTAAT<br>CTATCTAAACGGGGAACCTCTCTAGTAGACAATCCCGTGCTAAATTGTAGGACTTATA<br>TGCGTTCATATTATAG

CircRNA^{EGFP} templete (ePIE)	GGGAGACCCTCGACCGTCGATTGTCCACTGGTCAACAATAGATGACTTACAACTAAT<br>CGGAAGGTGCAGAGACTCGACGGGAGCTACCCTAACGTCAAGACGAGGGTAAAGA<br>GAGAGTCCAATTCTCAAAGCCAATAGGCAGTAGCGAAAGCTGCAAGAGAATGAAA<br>ATCCGTTGACCTTAAACGGTCGTGTGGGTTCAAGTCCCTCCACCCCCACGCCGGAA<br>ACGCAATAGCCGAAAAAACAAAAAACAAAAAACAAAACAAAATTAAAACAGCCT<br>GTGGGTTGATCCCACCCACAGGCCCATTGGGCGCTAGCACTCTGGTATCACGGTACC<br>TTTGTGCGCCTGTTTTATACCCCCTCCCCCAACTGTAACTTAGAAGTAACACACACC<br>GATCAACAGTCAGCGTGGCACACCAGCCACGTTTTGATCAAGCACTTCTGTTACCC<br>CGGACTGAGTATCAATAGACTGCTCACGCGGTTGAAGGAGAAAGCGTTCGTTATCC<br>GGCCAACTACTTCGAAAAACCTAGTAACACCGTGGAAGTTGCAGAGTGTTTCGCTC<br>AGCACTACCCCAGTGTAGATCAGGTCGATGAGTCACCGCATTCCCCACGGGCGACC<br>GTGGCGGTGGCTGCGTTGGCGGCCTGCCCATGGGGAAACCCATGGGACGCTCTAAT<br>ACAGACATGGTGCGAAGAGTCTATTGAGCTAGTTGGTAGTCCTCCGGCCCCTGAAT<br>GCGGCTAATCCTAACTGCGGAGCACACACCCTCAAGCCAGAGGGCAGTGTGTCGTA<br>ACGGGCAACTCTGCAGCGGAACCGACTACTTTGGGTGTCCGTGTTTCATTTTATTCC<br>TATACTGGCTGCTTATGGTGACAATTGAGAGATCGTTACCATATAGCTATTGGATTGG<br>CCATCCGGTGACTAATAGAGCTATTATATATCCCTTTGTTGGGTTTATACCACTTAGCT<br>TGAAAGAGGTTAAAACATTACAATTCATTGTTAAGTTGAATACAGCAAAGCCACCAT<br>GGTGAGCAAGGGCGAGGAGCTGTTCACCGGGGTGGTGCCCATCCTGGTCGAGCTG<br>GACGGCGACGTAAACGGCCACAAGTTCAGCGTGTCCGGCGAGGGCGAGGGCGATG<br>CCACCTACGGCAAGCTGACCCTGAAGTTCATCTGCACCACCGGCAAGCTGCCCGTG<br>CCCTGGCCCACCCTCGTGACCACCCTGACCTACGGCGTGCAGTGCTTCAGCCGCTA<br>CCCCGACCACATGAAGCAGCACGACTTCTTCAAGTCCGCCATGCCCGAAGGCTACG<br>TCCAGGAGCGCACCATCTTCTTCAAGGACGACGGCAACTACAAGACCCGCGCCGA<br>GGTGAAGTTCGAGGGCGACACCCTGGTGAACCGCATCGAGCTGAAGGGCATCGAC<br>TTCAAGGAGGACGGCAACATCCTGGGGCACAAGCTGGAGTACAACTACAACAGCC<br>ACAACGTCTATATCATGGCCGACAAGCAGAAGAACGGCATCAAGGTGAACTTCAAG<br>ATCCGCCACAACATCGAGGACGGCAGCGTGCAGCTCGCCGACCACTACCAGCAGA<br>ACACCCCCATCGGCGACGGCCCCGTGCTGCTGCCCGACAACCACTACCTGAGCACC<br>CAGTCCGCCCTGAGCAAAGACCCCAACGAGAAGCGCGATCACATGGTCCTGCTGG<br>AGTTCGTGACCGCCGCCGGGATCACTCTCGGCATGGACGAGCTGTACAAGTAAAAA<br>AAAAAAAAAAAAAAAAACGGCTATTATGCGTTACCGGCGAGACGCTACGGACTTA<br>AATAATTGAGCCTTAAAGAAGAAATTCTTTAAGTGGATGCTCTCAAACTCAGGGAA<br>ACCTAAATCTAGTTATAGACAAGGCAATCCTGAGCCAAGCCGAAGTAGTAATTAGTA<br>AGACCAGTGGACAATCGACGGATAACAGCATATCTAG
CircRNA$^{\text{E7-D2GFP}}$ templete (CPIEb)	TTATAATGTGGATGTATATAGGTTCTACATAAATGCCTAACGACTATCCCTTTGGGGA<br>GTAGGGTCAAGTGACTCGAAACGATAGACAACTTGCTTTAACAAGTTGGAGATATA<br>GTCTGCTCTGCATGGTGACATGCAGCTGGATATAATTCCGGGGTAAGATTAACGACC<br>TTATCTGAACATAATGCTACCGTTTAATATTGCGTCATTGGATTGGCAGCCAGACTGC<br>TGACTTACAACTAATCGGAAGGTGCAGAGACTCGACGGGAGCTACCCTAACGTCAA<br>GACGAGGGTAAAGAGAGAGTCCAATTCTCAAAGCCAATAGGCAGTAGCGAAAGCT<br>GCAAGAGAATGAAAATCCGTAAAAAACAAAAAACAAAAAACAAAACAAAATTAA<br>AACAGCCTGTGGGTTGATCCCACCCACAGGCCCATTGGGCGCTAGCACTCTGGTAT<br>CACGGTACCTTTGTGCGCCTGTTTTATACCCCCTCCCCCAACTGTAACTTAGAAGTA

	ACACACACCGATCAACAGTCAGCGTGGCACACCAGCCACGTTTTGATCAAGCACTT<br>CTGTTACCCCGGACTGAGTATCAATAGACTGCTCACGCGGTTGAAGGAGAAAGCGT<br>TCGTTATCCGGCCAACTACTTCGAAAAACCTAGTAACACCGTGGAAGTTGCAGAGT<br>GTTTCGCTCAGCACTACCCCAGTGTAGATCAGGTCGATGAGTCACCGCATTCCCCAC<br>GGGCGACCGTGGCGGTGGCTGCGTTGGCGGCCTGCCCATGGGGAAACCCATGGGA<br>CGCTCTAATACAGACATGGTGCGAAGAGTCTATTGAGCTAGTTGGTAGTCCTCCGGC<br>CCCTGAATGCGGCTAATCCTAACTGCGGAGCACACACCCTCAAGCCAGAGGGCAGT<br>GTGTCGTAACGGGCAACTCTGCAGCGGAACCGACTACTTTGGGTGTCCGTGTTTCA<br>TTTTATTCCTATACTGGCTGCTTATGGTGACAATTGAGAGATCGTTACCATATAGCTAT<br>TGGATTGGCCATCCGGTGACTAATAGAGCTATTATATATCCCTTTGTTGGGTTTATACC<br>ACTTAGCTTGAAAGAGGTTAAAACATTACAATTCATTGTTAAGTTGAATACAGCAAA<br>GCCACCATGGGACCAGCAGGACAGGCAGAACCAGATAGAGCCCACTACAACATCG<br>TGACCTTTTGCTGCAAGTGCGATGTGAGCAAGGGCGAGGAGCTGTTCACCGGGGT<br>GGTGCCCATCCTGGTCGAGCTGGACGGCGACGTAAACGGCCACAAGTTCAGCGTG<br>TCCGGCGAGGGCGAGGGCGATGCCACCTACGGCAAGCTGACCCTGAAGTTCATCTG<br>CACCACCGGCAAGCTGCCCGTGCCCTGGCCCACCCTCGTGACCACCCTGACCTACG<br>GCGTGCAGTGCTTCAGCCGCTACCCCGACCACATGAAGCAGCACGACTTCTTCAAG<br>TCCGCCATGCCCGAAGGCTACGTCCAGGAGCGCACCATCTTCTTCAAGGACGACGG<br>CAACTACAAGACCCGCGCCGAGGTGAAGTTCGAGGGCGACACCCTGGTGAACCGC<br>ATCGAGCTGAAGGGCATCGACTTCAAGGAGGACGGCAACATCCTGGGGCACAAGC<br>TGGAGTACAACTACAACAGCCACAACGTCTATATCATGGCCGACAAGCAGAAGAAC<br>GGCATCAAGGTGAACTTCAAGATCCGCCACAACATCGAGGACGGCAGCGTGCAGC<br>TCGCCGACCACTACCAGCAGAACACCCCCATCGGCGACGGCCCCGTGCTGCTGCCC<br>GACAACCACTACCTGAGCACCCAGTCCGCCCTGAGCAAAGACCCCAACGAGAAGC<br>GCGATCACATGGTCCTGCTGGAGTTCGTGACCGCCGCCGGGATCACTCTCGGCATG<br>GACGAGCTGTACAAGAAGCTTAGCCATGGCTTCCCGCCGGAGGTGGAGGAGCAGG<br>ATGATGGCACGCTGCCCATGTCTTGTGCCCAGGAGAGCGGGATGGACCGTCACCCT<br>GCAGCCTGTGCTTCTGCTAGGATCAATGTGTAGAAAAAAAAAAAAAAAAAAAAAAC<br>GGACTTAAATAATTGAGCCTTAAAGAAGAAATTCTTTAAGTGGATGCTCTCAAACTC<br>AGGGAAACCTAAATCTAGTTATAGACAAGGCAATCCTGAGCCAAGCCGAAGTAGTA<br>ATTAGTAAGTTAGCAGTCTGGCTGCCAATCCAAGATGTTTTCTTGGGTTAATTGAGG<br>CCTGAGTATAAGGTGACTTATACTTGTAATCTATCTAAACGGGGAACCTCTCTAGTAG<br>ACAATCCCGTGCTAAATTGTAGGACTTATATGCGTTCATATTATAG
CircRNA^{dRBD} templete (CPIEb)	TTATAATGTGGATGTATATAGGTTCTACATAAATGCCTAACGACTATCCCTTTGGGGA<br>GTAGGGTCAAGTGACTCGAAACGATAGACAACTTGCTTTAACAAGTTGGAGATATA<br>GTCTGCTCTGCATGGTGACATGCAGCTGGATATAATTCCGGGGTAAGATTAACGACC<br>TTATCTGAACATAATGCTACCGTTTAATATTGCGTCATTGGATTGGCAGCCAGACTGC<br>TGACTTACAACTAATCGGAAGGTGCAGAGACTCGACGGGAGCTACCCTAACGTCAA<br>GACGAGGGTAAAGAGAGAGTCCAATTCTCAAAGCCAATAGGCAGTAGCGAAAGCT<br>GCAAGAGAATGAAAATCCGTAAAAAACAAAAAACAAAAAACAAAACAAAATTAA<br>AACAGCCTGTGGGTTGATCCCACCCACAGGCCCATTGGGCGCTAGCACTCTGGTAT<br>CACGGTACCTTTGTGCGCCTGTTTTATACCCCCTCCCCCAACTGTAACTTAGAAGTA<br>ACACACACCGATCAACAGTCAGCGTGGCACACCAGCCACGTTTTGATCAAGCACTT<br>CTGTTACCCCGGACTGAGTATCAATAGACTGCTCACGCGGTTGAAGGAGAAAGCGT

	TCGTTATCCGGCCAACTACTTCGAAAAACCTAGTAACACCGTGGAAGTTGCAGAGT GTTTCGCTCAGCACTACCCCAGTGTAGATCAGGTCGATGAGTCACCGCATTCCCCAC GGGCGACCGTGGCGGTGGCTGCGTTGGCGGCCTGCCCATGGGGAAACCCATGGGA CGCTCTAATACAGACATGGTGCGAAGAGTCTATTGAGCTAGTTGGTAGTCCTCCGGC CCCTGAATGCGGCTAATCCTAACTGCGGAGCACACACCCTCAAGCCAGAGGGCAGT GTGTCGTAACGGGCAACTCTGCAGCGGAACCGACTACTTTGGGTGTCCGTGTTTCA TTTTATTCCTATACTGGCTGCTTATGGTGACAATTGAGAGATCGTTACCATATAGCTAT TGGATTGGCCATCCGGTGACTAATAGAGCTATTATATATCCCTTTGTTGGGTTTATACC ACTTAGCTTGAAAGAGGTTAAAACATTACAATTCATTGTTAAGTTGAATACAGCAAA GCCACCATGATCCACAGCGTGTTCCTGCTGATGTTCCTCCTGACCCCTACCGAGAGC AGAGTGCAGCCCACAGAGTCCATCGTGCGGTTTCCCAATATTACAAACCTGTGTCCC TTCGGGGAAGTGTTCAACGCCACAAGATTCGCTAGCGTGTATGCTTGGAATAGAAA AAGAATTTCCAACTGCGTGGCTGACTACAGCGTGCTGTACAACTCCGCTAGCTTCA GCACCTTCAAGTGCTATGGGGTGAGCCCCACCAAGCTGAACGATCTGTGCTTTACC AATGTGTATGCCGACAGCTTCGTGATTAGAGGGGATGAGGTCCGGCAGATCGCTCC CGGCCAAACCGGCAAGATCGCCGACTACAATTACAAGCTCCCCGATGACTTTACCG GCTGCGTGATCGCCTGGAATAGCAACAACCTGGACTCCAAGGTGGGCGGCAATTAC AATTACAGATACAGACTGTTTCGGAAGTCCAACCTGAAACCCTTCGAGCGGGATAT CTCCACCGAAATCTACCAAGCCGGGAGCAAGCCCTGTAACGGCGTGGAGGGCTTC AACTGCTACTTTCCCCTGCAGAGCTACGGCTTTCAGCCCACCAACGGCGTGGGCTA TCAGCCCTACAGAGTGGTCGTGCTCAGCTTCGAGCTGCTCCATGCTCCCGCTACCGT GTGCGGCCCCAAGAAATCCACCAACCTGGTGAAGAACAAGCGGGTGCAGCCCACC GAGAGCATCGTCAGATTCCCCAACATCACCAACCTGTGCCCCTTCGACGAGGTGTT CAACGCTACAAGATTCGCTAGCGTGTACGCCTGGAATAGAAAAAGAATTAGCAATT GTGTGGCTGATTATAGCGTCCTGTACAACTTCGCCCCCTTCTTCGCCTTCAAGTGCTA CGGCGTCTCCCCCACAAAGCTGAACGACCTGTGCTTCACCAACGTGTACGCCGATA GCTTTGTGATCAGAGGCAACGAAGTGAGCCAAATCGCCCCCGGGCAGACCGGCAA CATTGCCGACTACAACTACAAGCTGCCCGACGACTTCACCGGCTGCGTCATCGCTT GGAACAGCAATAAGCTGGACAGCAAGGTGGGCGGCAACTATAACTACCTCTACAGA CTGTTCAGAAAGAGCAACCTGAAGCCTTTCGAGCGGGACATCAGCACCGAGATTTA CCAAGCCGGCAACAAGCCCTGCAATGGCGTGGCTGGCTTCAACTGTTACTTCCCCC TGCGCAGCTACGGCTTCAGACCCACCTACGGCGTGGGCCATCAGCCCTACCGGGTG GTCGTGCTGTCCTTCGAGCTGCTGCACGCTCCCGCCACCGTGTGTGGCCCCAAGAA AAGCACCAATCTGGTCAAGAACAAGCATCACCATCACCATCACTGAAAAAAAAAA AAAAAAAAAAAACGGACTTAAATAATTGAGCCTTAAAGAAGAAATTCTTTAAGTGG ATGCTCTCAAACTCAGGGAAACCTAAATCTAGTTATAGACAAGGCAATCCTGAGCC AAGCCGAAGTAGTAATTAGTAAGTTAGCAGTCTGGCTGCCAATCCAAGATGTTTTCT TGGGTTAATTGAGGCCTGAGTATAAGGTGACTTATACTTGTAATCTATCTAAACGGGG AACCTCTCTAGTAGACAATCCCGTGCTAAATTGTAGGACTTATATGCGTTCATATTATA G
qPCR primers	
EGFP-qF	CCACATGAAGCAGCACGACTT
EGFP-qR	CGATGTTGTGGCGGATCTTGA
Circ-qF	GATCACTCTCGGCATGGACGA

Circ-qR	GCAGTCTATTGATACTCAGTC
RIG-1-qF	TGTGGGCAATGTCATCAAAA
RIG-1-qR	GAAGCACTTGCTACCTCTTGC
IL-6-qF	AGACAGCCACTCACCTCTTCAG
IL-6-qR	TTCTGCCAGTGCCTCTTTGCTG
TNFα-qF	CTCTTCTGCCTGCTGCACTTTG
TNFα-qR	ATGGGCTACAGGCTTGTCACTC
IFNβ-qF	CTTGGATTCCTACAAAGAAGCAGC
IFNβ-qR	TCCTCCTTCTGGAACTGCTGCA
β-actin-qF	CACCATTGGCAATGAGCGGTTC
β-actin-qR	AGGTCTTTGCGGATGTCCACGT
